# Characteristics of global naturopathic education, regulation, and practice frameworks: results from an international survey

**DOI:** 10.1186/s12906-021-03217-1

**Published:** 2021-02-18

**Authors:** J. M. Dunn, A. E. Steel, J. Adams, I. Lloyd, N. De Groot, T. Hausser, J. Wardle

**Affiliations:** 1 University of Technology Sydney, 15 Broadway, Ultimo, NSW 2007 Australia; 2 Australian Research Centre in Complementary & Integrative Medicine (ARCCIM), School of Public Health, University of Technology Sydney, 15 Broadway, Ultimo, NSW 2007 Australia; 3 World Naturopathic Federation, 20 Holly St, Suite, Toronto, 200 Canada; 4 Canadian College of Naturopathic Medicine, 1255 Sheppard Ave East, Toronto, Ontario Canada; 5 National Centre for Naturopathic Medicine, Southern Cross University, Military Road, Lismore, NSW 2480 Australia

**Keywords:** Complementary medicine, Education, Naturopathy, Regulation

## Abstract

**Background:**

This descriptive study provides the first examination of global naturopathic education, regulation and practice frameworks that have potential to constrain or assist professional formation and integration in global health systems. Despite increasing public use, a significant workforce, and World Health Organization calls for national policy development to support integration of services, existent frameworks as potential barriers to integration have not been examined.

**Methods:**

This cross-sectional survey utilized purposive sampling of 65 naturopathic organisations (educational institutions, professional associations, and regulatory bodies) from 29 countries. Organizational representatives completed an on-line survey, conducted between Nov 2016 – Aug 2019. Frequencies and cross-tabulation statistics were analyzed using SPSSv.25. Qualitative responses were hand-coded and thematically analysed where appropriate.

**Results:**

Sixty-five of 228 naturopathic organizations completed the survey (29% response rate) from 29 of 46 countries (63% country response rate). Most education programs (68%) were delivered via a national framework. Higher education qualifications (60%) predominated. Organizations influential in education were professional associations (75.4%), particularly where naturopathy was unregulated, and accreditation bodies (41.5%) and regulatory boards (33.8%) where regulated. Full access to controlled acts, and to health insurance rebates were more commonly reported where regulated. Attitude of decision-makers, opinions of other health professions and existing legislation were perceived to most impact regulation, which was globally heterogeneous.

**Conclusion:**

Education and regulation of the naturopathic profession has significant heterogeneity, even in the face of global calls for consistent regulation that recognizes naturopathy as a medical system. Standards are highest and consistency more apparent in countries with regulatory frameworks.

**Supplementary Information:**

The online version contains supplementary material available at 10.1186/s12906-021-03217-1.

## Background

Naturopathy is a philosophically-defined, distinct system of traditional and complementary medicine (T&CM), rooted in traditional European and North American practices and recognized by the World Health Organization (WHO) [1]. Naturopaths are not defined by their therapeutic tools, but provide primary contact care in accordance with underlying principles: first do no harm (*primum non nocere*); healing power of nature (*vis medicatrix naturae*); treat the cause (*tolle causam*); treat the whole person (*Tolle totum*); doctor as teacher (*docere*); disease prevention and health promotion; wellness [2].

Consumers globally are increasingly consulting naturopathic practitioners to enhance well-being, for common health complaints, and serious chronic conditions [3], with exponential growth in use and expenditure over the past 25 years [4, 5]. The recent Australian prevalence rate for naturopathic consultations was 6.2% [6], and in Switzerland, the consultation prevalence rate was similar 7.7% (7.2–8.2), with use markedly increasing when patients had access to health insurance rebates [7].

Naturopaths constitute an appreciable part of the health care sector [8], and are globally regulated as naturopathic technicians, licensed naturopaths and naturopathic doctors (ND) [9]. The *World Naturopathic Federation* (WNF) estimates 75,000–100,000 naturopathic practitioners practice in 81 countries, with over 90 educational institutions providing naturopathic training [10], and 20 naturopathic research institutions globally [11].

Naturopathic education and regulation have been examined primarily in single country studies. These studies have examined the challenges of implementing accreditation and regulation [12], descriptive studies of accreditation standards and training competencies [13, 14], descriptive studies on the scope of naturopathic regulation in individual countries [15–17] and comparison of regulation between one country and another [18, 19] as well as comparisons between naturopathic regulation and regulation of other professions in single countries [20]. There have also been studies of the various limitations of existing regulatory models in South Africa [21] and Australia [22], as well as examination of the impact of disharmonious regulation across the European Union [23, 24].

Despite increasing public use, a significant naturopathic workforce, and calls by the WHO for national policy development for integration of services, existent frameworks as potential barriers to integration have not been examined at an international level. In response to this significant research gap, this paper reports findings from a first global examination of regulation, education, and practice frameworks that may impact naturopathic professional formation and integration into health care systems.

## Methods

### Study design

This descriptive study reports results from analysis of a cross-sectional survey of 65 naturopathic organizations (including naturopathic educational institutions, professional associations, and regulatory bodies) from 29 countries and is the initial phase of a larger project examining education and regulation of the global naturopathic profession. The survey instrument was developed in conjunction with members of the WNF Professional Mapping Committee.

### Setting

Participants completed an online survey, in the English language, through the SurveyGizmo platform. Recruitment and data collection occurred between November 2016 and August 2019 and collection dates can be viewed in Supplementary File 2. An extended survey timeframe was necessary as new organizations emerged.

### Participants

Global naturopathic organizations were recruited using purposive sampling with 228 organizations from 46 countries invited to participate. Naturopathic organizations were identified by WNF and complemented by additional internet searches. Organizational leaders were invited by email to participate in this survey where the organization was primarily focused on naturopathy, met WHO training guidelines (educational institutions), or provided or accepted highest national education standards, as identified by WNF. A formal invitation and research information sheet was provided with link to the survey. Participants provided consent by pre-reading the research information sheet before starting the survey. The survey took approximately 60 min to complete. A total of twenty follow up emails were sent to participants over 34 months. Where English skills impacted the ability to complete the survey, participants were requested to collaborate with an English-speaking colleague, or to contact the researcher for translation assistance through WNF. No translation assistance was requested.

### Measures

The survey consisted of 59 items relating to six domains – *(1) organization demographics, (2) education characteristics, (3) organizational influence on education, (4) regulatory characteristics, (5) perception of regulation, (6) consultation reimbursement characteristics.* Definitions of key terms were provided. Survey logic was used to customize questions to organizational and regulation type. The questionnaire was piloted on a convenience sample of organizations from five countries. Minor changes were made to the questionnaire based on respondent feedback.

The items, scales and open form questions used in the survey are described in this section. Most survey items were multiple choice (69%), 12% were seven-point rating scales, and 42% provided comment. Open form items accounted for 19% of the questionnaire. The survey can be found in Supplementary File 1.

#### Organization demographics

Organizational demographic items measured organization type [education institution, professional association, or regulatory body], WHO training guidelines supported [min 1500 h, min 2 years in length, min 400 h supervised clinical program], country, jurisdiction, leader characteristics and provided organizational contact details.

#### Education characteristics

Education characteristic items measured institutional attributes [profit, not for profit or state], qualifications offered [year first offered, title, undergraduate, or postgraduate, duration], educational framework [and framework name] used to guide qualification development.

#### Organizational influence on education

Items measured perceived influence of organizations [educational institutions, national and regional professional associations, third-party funders, national and regional government departments, industry, multi-national body and accreditation body] on education, delivery, content, admission requirements, tuition fee schedule, clinical supervisor/student ratio, total course hours, program duration, learning outcomes, minimum qualifications, clinical and research experience of classroom teachers and clinical supervisors. Items identified and measured organizations responsible for external audits and types of audit undertaken.

#### Regulatory characteristics

Regulatory characteristic items measured organizations involved, regulation type [‘no regulation’, ‘title protection’, ‘defined scope of practice’, or ‘other’ regulation], date regulation was first implemented, jurisdiction, restricted or controlled acts, professional-entry registration requirements, regulatory board characteristics, factors that determine practices, organizations that impact standards, regulation of, and access to natural health products. Items measured access to private insurance, public funding, and hospital privileges.

#### Perception of regulation

Open form questions provided multiple comment by organizations on perceptions held about regulation, factors impacting regulation, and organizations supporting or opposing naturopathic regulation. A seven-point rating scale plus not applicable option measured perceived impact of regulation on education and vice versa, and the degree to which reimbursement was perceived tied to regulation, influenced integration and access to naturopathic services.

#### Consultation reimbursement characteristics

Items measured payment methods for naturopathic services [direct payment by patient, government funded, private health insurance reimbursement, other third-party funder] and payment restrictions [consultation costs, naturopathic prescription products, in-office treatments (e.g. acupuncture, manual therapy)]. Impact of perceived factors [malpractice insurance, license, practitioner characteristics, condition type, practitioner qualification, referral from medical practitioner, type of therapies provided] on patient access to reimbursement were measured, as was perceived degree to which reimbursement costs associated with naturopathic care were tied to regulation in the country.

### Data analysis

Descriptive statistics were tabulated as frequencies and percentages using IBM SPSS Statistics Standard Edition Version 25 and cross-tabulations calculated. Qualitative open form questions were colour coded by hand and thematically analysed where appropriate, and reported by frequency of themes, named organizations and relevant organization-specific comments were summarized and used to elaborate quantitative data. Prior to analysis, raw data were screened for incomplete responses or double ups. Partially completed (104) and disqualified (30) responses were removed from analysis. Medians were calculated for rating scales. Data was analysed by type of regulation as compared to country in order to examine the impact of method of regulation on regulatory framework characteristics, naturopathic practices, treatment reimbursements and integration into health systems. Table 8 analysed by country is also available as Supplementary File 3 Country Restricted Acts.

## Results

### Organizational demographics

A total of 65 responses were analyzed (29% response rate) from 29 of 46 countries (63%). All six WHO world regions were represented. The total study population by world region, country participation and organizational type can be found in Table 1.

**Table 1:** Total study population by world region, country participation and organizational type (Source: World Health Organization, 2019)

World Region	Participation by country (***n=29***) and organization type	Non-participation by country (***n=17***)
Africa	Congo DR^a^, South Africa^b,c^, Zambia^c^	Kenya
Americas	Brazil^a,b^, Canada^a,b,c^, Chile^a^, Mexico^a^, Peru^a^, Puerto Rico^a,b^, USA^a,b,c^, Uruguay^a,b^, Venezuela^b^	Argentina, Colombia, Costa Rica, Ecuador, El Salvador, Honduras, Panama, Paraguay
Eastern Mediterranean	Egypt^a^	Morocco
Europe	Belgium^a,b^, Cyprus^a^, Czech Republic^a,b^, France^a,b^, Germany^a^, Italy^a,b^, Portugal^a^, Slovenia^a,b^, Spain^a^, Sweden^a^, Switzerland^a^, United Kingdom^a,b^	Denmark, Iceland, Ireland, Norway
South East Asia	Nepal^b^	India
Western Pacific	Australia^a,b,c^, Hong Kong^a^, New Zealand^a,b^	Japan, Malaysia

^a^. Professional association; ^b^. Educational institution; ^c^. Regulator of naturopathy

Respondents primarily represented professional associations (53.8%) (*n=35)* followed by educational institutions (38.5%) (*n=25*) and regulatory boards (7.7%) (*n=5*). Most professional associations and regulatory boards reported national jurisdiction (82.5%) (*n=33*), with (58.5%) (*n=38*) of total respondents reporting one national professional association. In Australia and France up to seven national professional associations were reported, and in Germany 10 or more.

### Characteristics of naturopathic education

Of 65 respondent organizations that completed the survey, 63.1% (*n=41)* reported their countries met all three WHO training guidelines for naturopathic medicine. Most educational institutions (80%) (*n=20)* reported the program length exceeded two-year recommended minimums, with almost half reporting programs of four-years in length (*n=12*) (48%). Higher education programs and qualifications (60%) (*n=15*) predominated. Table 2 represents the characteristics of educational programs, institutions, and auditing agencies that impact naturopathic education, reported by educational institutions in this study.

**Table 2:** Characteristics of global naturopathic education, programs, and institutions

	N	%
**Characteristics of naturopathic education according to WHO guidelines (*n*=65)**		
Minimum 1500 contact hours	55	84.6
Minimum 2 years length	49	75.4
Minimum 400 supervised clinical hours	47	72.3
All three guidelines met	41	63.1
No guidelines met	5	7.7
**Characteristics of naturopathic educational programs (*n*=25)**		
Program length		
*2 years*	4	16.0
*3 years*	8	32.0
*4 years*	12	48.0
Program and Qualification type		
*Vocational (Diploma or unspecified qualification level)*	10	40.0
*Higher education*	15	60.0
*Undergraduate bachelor’s degree [Australia, Brazil, NZ]*	6	24.0
*Postgraduate qualification [Canada, Puerto Rico, South Africa, UK, USA]*	9	36.0
Qualification delivered by national qualifications’ framework		
^a^*Yes*	17	68.0
*No*	8	32.0
**Characteristics of naturopathic educational institutions (n=25)**		
*For profit*	15	60.0
*Not for profit*	9	36.0
*State*	1	4.0
Year educational institution first offered naturopathic program		
*1956–1975*	4	16.0
*1976–1995*	5	20.0
*1996–2015*	14	56.0
**Characteristics of program audits (n=25)**		
Schools reporting some type of external audit		
*Yes*	19	76.0
*No*	6	24.0
Organizations responsible for external audits^b^		
*Government*	11	30.6
*Private*	0	0.0
*Professional association*	12	33.3
*Accrediting body*	11	30.6
*Other*	2	5.6
External audit type^c^		
*Governance/quality assurance*	18	19.8
*Course content, delivery, and assessment*	27	29.7
*Clinical processes*	20	22.0
*Financial*	17	18.7
*Other*	9	9.9
**Characteristics of organizational influence on naturopathic education (n=65)**		
Perceived influence of organizations (other than educational institution) on delivery and content of education		
*National Professional Association*	49	75.4
*Regional Professional Association*	13	20.0
*Accreditation Body*	27	41.5
*Regulatory Board*	22	33.8
*National Government*	18	27.7
*Regional Government*	6	9.2
*Other Health Professionals*	14	21.5
*Third-Party Funders*	13	20.0
*Multi-National Body*	8	12.3

^a^Included Italy based on UNI ISO standard; ^b.^(n=19 [36 responses]); ^c.^(n=24 [91 responses])

Most naturopathic educational institutions were private (96%) (*n=24)*, and most were for-profit establishments (60%) (*n=15).* While some programs were offered as early as 1956, most formal programs (56%) (*n=14)* were established in the period between 1996 and 2015. Approximately two thirds (68%) (*n=17)* of naturopathic educational institutions reported program delivery via a national qualifications’ framework (either general or naturopathic-specific – see Tables 2 and 3), although Italy’s was based on a national ISO standard.

**Table 3:** National qualifications frameworks reported by educational institutions and accreditation bodies by total organizations

Country	National Qualifications Framework	Accreditation Body
Australia	Tertiary Education Quality Standards Agency [TESQA]	Un-named
Brazil	Ministry of Education and Culture [MEC]	Not applicable
Canada	The Council on Naturopathic Medical Education [CNME], Post-secondary Education Quality Assessment Board [PEQAB] or other regional state educational institution accreditation requirements	The Council on Naturopathic Medical Education [CNME]
France^b^	Not applicable	Naturopathic education consortium
Germany	Unrepresented	Regional Health Offices
Italy^a^	International Organisation for Standardization [ISO] Ente Nazionale Italiano di Unificazione [UNI] 11,491,	ACCREDIA
Mexico	Unrepresented	Secretaria de Educación Pública [SEP])
Nepal	Council for Technical Education and Vocational Training [CTEVT]	Council for Technical Education and Vocational Training [CTEVT]
New Zealand (NZ)^b^	NZ Qualifications Authority [NZQA]	Natural Health Practitioners of NZ
Portugal	Unrepresented	Central Administration of the Health System [ACSS]
Puerto Rico	CNME, Consejo de Educación de Puerto Rico	CNME
South Africa	Council of Higher Education [CHE]	Statutory regulatory body
Switzerland	Unrepresented	Organisation der Arbeitswelt AlternativMedizin Schweiz [OdA-AM]
Sweden^b^	Unrepresented	Naturopathic professional association
United Kingdom (UK)^b^	Framework for Higher Education Qualifications [FHEQ],	Complementary and Natural Healthcare Council [CNHC], General Naturopathic Council [GNC]
USA	CNME, with regional state educational institution accreditation requirements	CNME

^a^ISO standard not national qualification framework; ^b^Self-accreditation organizations

### Organizational influence on education

#### Program content and delivery

Table 2 also represents organizations independent of educational institutions, reported by all respondents to influence naturopathic program content and delivery. Of 65 respondents, most reported national professional associations (75.4%) (*n=49*), followed by accreditation bodies (41.5%) (*n=27);* regulatory boards (33.8%) (*n=22*); national government departments (27.7%) (*n=18)* and other health professions (21.5%) (*n=14*) as stakeholders influencing naturopathic program content and delivery. Where naturopathy was unregulated (*n=28)*, national professional associations (89.2%) (*n=25*) followed by national government departments (42.9%) (*n=12*) were most frequently reported to influence program content and delivery. Where regulated, accreditation bodies and regulatory boards were most commonly reported as influential in influencing naturopathic program content and delivery. The presence of multiple professional associations in Australia was reported to create complexity, and hinder educational and professional progress, as the following representative quote from the free-text section demonstrates:

“*There are a number of professional associations and these associations often differ in their views causing disunity. This creates challenges for* [NAME OF COLLEGE] *as we attempt to develop courses which prepare students for future practice, as the profession can disagree on what that practice should look like”.* (Australian educational institution).

Naturopathic educational institutions were perceived to have significant influence on course, curriculum, and faculty characteristics. Educational institutions were perceived to have ‘moderate to high’ influence on course tuition fees, delivery method and faculty characteristics – teacher and clinical supervisor qualifications, clinical and research experience across all regulation types. Accreditation bodies were seen to have influence in course content, admission requirements and clinical supervisor/student ratio in some regulated jurisdictions.

#### Program audit

Most naturopathic educational institutions (76%) (*n=19)* reported some form of external audit by multiple organizations. Educational institutions most frequently reported program audits by professional associations (33.3%) (*n=12*), followed by government (30.5%) (*n=11)* and accreditation bodies (30.5%) (*n=11)* (see Table 2). Professional association audits were commonly reported where naturopathy was unregulated, and accreditation body and government audits were more common where the profession was regulated. Government audits were also reported when programs were delivered via a national framework regardless of regulatory status. Table 3 also represents reported accreditation bodies.

Of the 91 external audits reported by educational institutions (*n=24)* most were focused on content delivery and assessment (29.7%) (*n=27*), followed by clinical processes (22%) (*n=20*), and governance/quality assurance (19.8%) (*n=18*). Governance/quality assurance, course content, delivery and assessment, clinical processes and financial audits were commonly reported by educational institutions that delivered programs via a national qualifications’ framework. However, there was significant heterogeneity in how this was applied.

In Australia, where naturopathy was reported unregulated, a respondent reported national qualification framework compliance audits related to optimization of student outcomes, that is, completion of a program, as opposed to meeting professional standards. A respondent from Italy reported ‘other’ audit type by the UNI international standards organization (ISO). Respondents from France reported self-regulating organizations undertook governance/quality assurance, course content, delivery, and assessment audits. Respondents from Uruguay and Belgium reported self-regulating organization audit of course content and assessment, and by Belgium additional clinical and independent financial audit. Auditing was not reported in following unregulated jurisdictions - Czech Republic, Nepal, Slovenia, and Venezuela, nor in the UK where voluntary regulation was reported.

### Characteristics and perceptions of regulation

#### Regulation type

Regulation of naturopathy was reported to predate 1949 in Canada, Germany, and USA. There was significant heterogeneity of regulatory types, with organizations sometimes reporting more than one type (see Table 4). The most commonly reported regulatory status was ‘no regulation’ (43.1%) (*n=28*), followed by ‘defined scope of practice’ (41.5%) (*n=27*), ‘title protection’ (27.7%) (*n=18*) and ‘other’ type of regulation (10.8%) (*n=7*). Voluntary self-regulation was reported in Australia, Cyprus, UK, and Sweden. ‘*Heilpraktiker*’ regulation was reported in Germany, while in Switzerland regulation was by federal degree. In Congo DR, naturopathy was regulated under 1950 traditional/natural medicine legislation and the respondent from Italy reported regulation by voluntary national standardization (ISO) mechanisms.

**Table 4:** Regulation type/s reported by country

Country	No Regulation	Title Protection	Defined Scope of Practice	Other Regulation
Australia	*			*
Belgium^a^				
Brazil	*	*		
Canada		*	*	
Chile		*	*	
Congo DR			*	
Cyprus				*
Czech Republic	*			
Egypt^a^				
France	*			
Germany		*		*
Hong Kong^a^				
Italy			*	
Mexico			*	
Nepal	*	*	*	
New Zealand	*			
Peru	*			
Portugal		*	*	
Puerto Rico			*	
Slovenia	*			
South Africa		*	*	
Spain	*			
Switzerland	*	*	*	*
Sweden			*	
United Kingdom				*
USA	*	*	*	
Uruguay	*			
Venezuela	*			
Zambia	*			

^a^ Missing data – Belgium, Egypt, Hong Kong, State [USA] which did not indicate regulation type

‘Title protection’ and ‘defined scope of practice’ were not always tied to professional registration/licensing. Furthermore, the implementation of national policy at a state level was not always consistent, with each sub-national jurisdiction in Canada, Switzerland and the United States reported to apply their own rules and regulations.

#### Standards setting

Regulatory boards were reported most influential in setting standards when naturopathy was regulated by a ‘title protection’ mechanism (77.7%) (*n=14*) and/or a ‘defined scope of practice’ mechanism (74%) *(n=20*). Professional associations were reported responsible for setting standards when naturopathy was unregulated (75%) (*n=21*), and when ‘other’ types of regulation (85.7%) (*n=6*) existed. Enforcing standards/guidelines followed the same structure, as did requesting changes to standards, although, educational institutions were also reported influential in the latter. Educating practitioners about standards and guidelines generally involved all three organizations – educational institutions, regulatory boards, and national professional associations.

#### Accreditation and regulatory frameworks

Accreditation bodies were reported by 41.5% respondents (*n=*27), with 33.8% (*n=*22) reporting regulatory boards. These bodies were not always distinct organizations. Respondents from USA, Canada, Mexico, and Italy reported separate and independent accreditation and regulation/licensing boards for the naturopathic profession, although Nepal, Portugal, Switzerland, and South Africa had dual purpose boards (i.e. for both accreditation and regulation). The regulatory board was described by a respondent from Canada as being multi-purposes, and responsible for investigating and disciplining registrants, overseeing licensure, setting standards for a range of certifications, policies for emergency medicine and continuing education.

Professional entry requirements were globally inconsistent. Respondents from Canada, Chile, Congo DR, Nepal, Puerto Rico, South Africa, Switzerland, and USA (57.6%) (*n=19*), reported requirement for completion of a program from a specific naturopathic institution, with successful completion of a regulatory board examination. However, only respondents from Canada, Puerto Rico, and Switzerland, consistently reported this method (see Table 5). Direct entry into the profession based on completion of a specific qualification with no requirement to graduate from a naturopathic institution was reported by respondents from Congo DR, Italy, Nepal, Sweden, and UK. In Germany professional entry was based on successful completion of a board examination without any other specific qualification, with the professional association from Germany reporting that ‘*Heilpraktiker’* held a high level of self-responsibility concerning education and practice. Board examinations were reported not required in Brazil, and Mexico, where ‘title protection’ or ‘defined scope of practice’ were reported, and were one of multiple methods used in Chile, Congo DR, Nepal, and South Africa. Voluntary regulation was reported to not require professional entry examinations in Australia, Cyprus, and UK.

**Table 5:** Characteristics of global regulation of naturopathic practitioners

NATUROPATHIC REGISTRATION/LICENSING CHARACTERISTICS	Total (*n*=33)^a^		No regulation (*n*=28)^b^		Title protection (*n*=18)^b^		Defined scope of practice (*n*=27)^b^		Other regulation (*n*=7)^b^	
	N	%	N	%	N	%	N	%	N	%
**Profession entry requirement to be registered**										
*Direct entry based on qualification completion only*	6	18.2	1	3.6	1	5.6	5	18.5	1	14.3
*Direct entry based on qualification from a naturopathic education institution*	10	30.3	1	3.6	4	22.2	6	22.2	4	57.1
*Entry based on completion from naturopathic institution plus board examination*	19	57.6	2	7.1	13	72.2	19	70.3	1	14.3
*Entry based on success in board examination without any specific qualifications*	2	6.1	1	3.6	2	11.1	1	3.7	1	14.3
*Other*	4	12.1	1	3.6	2	11.1	3	11.1	1	14.3
**Organization that administers board examination for naturopathic regulation**										
*Government Department or Agency*	2	6.1	1	3.6	2	11.1	2	7.4	0	0.0
*Delegated Authority on Behalf of Government*	3	9.1	1	3.6	3	16.7	3	11.1	1	14.3
*Independent Academic Accreditation Agency*	2	6.1	0	0.0	1	5.6	2	7.4	0	0.0
*Naturopathic Educational Institution*	0	0.0	0	0.0	0	0.0	0	0.0	0	0.0
*Naturopathic Organization Independent of Naturopathic Institution*	7	21.2	0	0.0	5	27.8	7	25.9	0	0.0
*Other*	5	15.1	0	0.0	2	11.1	5	18.5	0	0.0
**Requirements to sit board naturopathic registration/licensing examination**										
*No Entry Requirements*	1	3.0	0	0.0	0	0.0	1	3.7	0	0.0
*Entry Based on Experience or Equivalence Qualifications*	2	6.1	1	3.6	2	11.1	2	7.4	0	0.0
*Direct Entry Based on Qualification (no Institutional Accreditation required)*	1	3.0	0	0.0	1	5.6	1	3.7	0	0.0
*Direct Entry Based on an Accredited Institution only*	14	42.4	1	3.6	9	50.0	14	51.9	1	14.3
**Organization responsible for naturopathic registration/licensing approval**										
*Government official (*e.g. *Minister of Health)*	14	42.4	3	10.7	8	44.4	11	40.7	4	57.1
*A committee or board*	2	6.1	0	0.0	0	0.0	2	7.4	0	0.0
*An existing regulatory body for other health professions*	4	12.1	0	0.0	2	11.1	4	14.8	0	0.0
*An existing regulatory body for naturopathy*	7	21.2	0	0.0	5	27.8	6	22.2	1	14.3
*Other*	6	18.2	0	0.0	3	16.7	4	14.8	2	28.6
**Presence of naturopathy specific regulatory board**										
*Yes*	21	63.6	2	7.1	13	72.2	19	70.4	4	57.1
*No*	11	33.3	0	0.0	4	22.2	7	25.9	3	42.9
*Unsure*	1	3.0	1	3.6	1	5.6	1	3.7	0	0.0
**Characteristics of regulatory board for naturopathy**										
*Standalone organization with no government affiliation*	6	18.2	0	0.0	2	11.1	4	14.8	2	28.6
*Independent organization affiliated with government*	9	27.3	0	0.0	3	16.7	8	29.6	2	28.6
*Independent organization affiliated with a professional association*	2	6.1	2	7.1	2	11.1	2	7.4	1	14.3
*Government department*	13	39.4	1	3.6	10	55.6	12	44.4	1	14.3
*Professional association*	2	6.1	0	0.0	1	5.6	0	0.0	1	14.3
**Naturopathic regulation sole purpose of regulatory organization**										
*Yes*	18	54.5	2	7.1	9	50.0	15	55.6	5	71.4
*No*	10	30.3	0	0.0	7	38.9	9	33.3	1	14.3
*Unsure*	4	12.1	1	3.6	2	11.1	2	7.4	1	14.3
**Composition of committee of regulatory board**										
*Naturopaths/Naturopathic practitioners*	24	72.7	3	10.7	15	83.3	22	81.5	4	57.1
*Public/community representatives*	20	60.6	2	7.1	13	72.2	19	70.4	2	28.6
*Public servants*	3	9.1	0	0.0	2	11.1	2	7.4	1	14.3
*Government officials*	13	39.4	1	3.6	8	44.4	12	44.4	2	28.6
*Independent representatives of other health professions*	9	27.3	3	10.7	7	38.9	8	29.6	2	28.6
*Representatives of other health professional bodies (*e.g. *professional associations)*	7	21.2	1	3.6	3	16.7	6	22.2	1	14.3
*Representatives of naturopathic professional associations*	13	39.4	2	7.1	7	38.9	9	33.3	6	85.7
*Representatives of naturopathic educational institutions*	8	24.2	2	7.1	3	16.7	6	22.2	4	57.1

^a.^Data restricted to 33 respondents who reported regulation by ‘title protection’, ‘defined scope of practice’ or ‘other’. ^b.^ Some respondents reported multiple regulation types including ‘no regulation’ which is reported

Board examinations were administered by organizations independent of naturopathic educational institutions. Respondents from Puerto Rico and USA reported the *North American Board of Naturopathic Examiners* ascertained eligibility and administered licensing/registration examination, NPLEX. Regulators in Canada and South Africa were reported to undertake this function, and government departments in Chile and Nepal. Ability to sit board examinations were based on graduation from an accredited program in Canada, Puerto Rico, South Africa, Switzerland, and USA, with assessment of prior learning and recognition of substantial equivalency reported in Canada.

Licensing was mostly reported the responsibility of a government entity. Naturopathic specific regulatory boards were reported to be commonplace in jurisdictions with ‘title protection’ (72.2%) (*n=13*) and ‘defined scope of practice’ (70.3%) (*n=19*), although, naturopathic regulation was not always the boards’ sole purpose. The composition of regulatory boards varied according to regulation - where naturopathy had ‘title protection’ and/or ‘defined scope of practice’ the regulatory board comprised of naturopaths, independent community representatives and government officials. In contrast, where ‘other’ type of regulation was reported, the regulatory board was reported to comprise of mostly representatives from naturopathic professional associations, educational institutions and practicing naturopaths (see Table 5).

#### Regulation of naturopathic practices and access to tools of trade

Access to ‘tools of trade’ were not always freely available (see Table 6). Over the counter consumer access to botanical medicines was reported by 53.8% (*n=35*) respondents, nutraceuticals by 52.3% (*n=34*) and homeopathic medicines by 47.7% (*n=31*). Requirement for prescription, behind the counter, and health professional only access, were reported, potentially impacting naturopathic practice favorably or unfavorably depending on the definition of health professional, in some unregulated regions (see Table 7).

**Table 6:** Characteristics of natural product regulation and naturopathic ‘tools of trade’

ACCESS TO NATURAL PRODUCTS (n=65)		
Botanical medicines	N	%
*Restricted and requires a prescription*	11	16.9
*Access to health professional only*	8	12.3
*Restricted but available to consumers (*i.e. *behind the counter)*	13	20.0
*Freely available to consumers (*i.e. *over the counter)*	35	53.8
*Not available at all*	3	4.6
**Nutraceuticals**		
*Restricted and requires a prescription*	7	10.8
*Access to health professional only*	6	9.2
*Restricted but available to consumers (*i.e. *behind the counter)*	12	18.5
*Freely available to consumers (*i.e. *over the counter)*	34	52.3
*Not available at all*	2	3.1
**Homeopathics**		
*Restricted and requires a prescription*	9	13.8
*Access to health professional only*	12	18.5
*Restricted but available to consumers (*i.e. *behind the counter)*	11	16.9
*Freely available to consumers (*i.e. *over the counter)*	31	47.7
*Not available at all*	3	4.6
**Other**		
*Restricted and requires a prescription*	7	10.8
*Access to health professional only*	6	9.2
*Restricted but available to consumers (*i.e. *behind the counter)*	4	6.2
*Freely available to consumers (*i.e. *over the counter)*	8	12.3
*Not available at all*	3	4.6

**Table 7:** Limitation of naturopathic tools of trade reported by country

Country	Botanical Medicines	Nutraceuticals	Homeopathics
Australia			
*Restricted and requires prescription*	*	*	
*Access to health professionals only*	*	*	
Brazil			
*Restricted and requires prescription*	*	*	*
*Access to health professionals only*	*	*	*
Chile			
*Restricted and requires prescription*			*
*Access to health professionals only*			*
France			
*Restricted and requires prescription*	*		*
*Access to health professionals only*			*
Mexico			
*Restricted and requires prescription*	*	*	*
*Access to health professionals only*	*	*	*
Nepal			
*Restricted and requires prescription*	*	*	
*Access to health professionals only*	*	*	
Slovenia			
*Restricted and requires prescription*			
*Access to health professionals only*			*
UK			
*Restricted and requires prescription*	*	*	
*Access to health professionals only*	*		*
Uruguay			
*Restricted and requires prescription*			*
*Access to health professionals only*			*

Full access to restricted or controlled acts were more frequently reported where the profession was regulated (see Table 8). Organizations from Canada, USA, and Congo DR reported full access to a range of 14 potentially restricted acts (see supplementary information). Regulation did not always provide full access to the naturopathic scope of practice, with practices in Chile, Portugal and South Africa being restrictive relative to other regulated regions.

**Table 8:** Access by naturopaths to controlled or restricted acts^c^

	No Regulation (*n*=28)^b^	Title protection (*n*=18)^b^	Defined Practice (*n*=27)^b^	Other (n=7)^b^	No Regulation (*n*=28)^b^	Title protection (*n*=18)^b^	Defined Practice (*n*=27)^b^	Other (*n*=7)^b^
	N (%)	N (%)	N (%)	N (%)	N (%)	N (%)	N (%)	N (%)
	**Ordering pathology/ laboratory tests and scans**				**General physical examination**			
*Full Access*	3 (10.7)	11 (61.1)	12 (44.4)	2 (28.5)	17 (60.7)	16 (88.8)	24 (88.8)	4 (57.1)
*Limited Access*	10 (35.7)	6 (33.3)	14 (51.8)	3 (42.8)	2 (7.1)	1 (5.5)	2 (7.4)	2 (28.5)
*Permitted but NOT in Curriculum*	0 (0.0)	0 (0.0)	0 (0.0)	1 (14.2)	3 (10.7)	0 (0.0)	0 (0.0)	0 (0.0)
*Not Allowed*	15 (53.5)	1 (5.5)	1 (3.7)	1 (14.2)	6 (21.4)	1 (5.5)	1 (3.7)	0 (0.0)
*Unsure*	0 (0.0)	0 (0.0)	0 (0.0)	0 (0.0)	0 (0.0)	0 (0.0)	0 (0.0)	1 (14.2)
	**Obstetrics/ Maternity Care**				**Acupuncture**			
*Full Access*	0 (0.0)	4 (22.2)	6 (22.2)^a^	0 (0.0)	6 (21.4)	12 (66.6)	16 (59.2)	2 (28.5)
*Limited Access*	6 (21.4)	11 (61.1)	13 (48.1) ^a^	3 (42.8)	2 (7.1)	3 (16.6)	5 (18.5)	0 (0.0)
*Permitted but NOT in Curriculum*	4 (14.2)	0 (0.0)	1 (3.7) ^a^	0 (0.0)	4 (14.2)	0 (0.0)	1 (3.7)	4 (57.1)
*Not Allowed*	17 (60.7)	3 (16.6)	6 (22.2) ^a^	2 (28.5)	16 (57.1)	3 (16.6)	3 (11.1)	0 (0.0)
*Unsure*	1 (3.5)	0 (0.0)	0 (0.0)	2 (28.5)	0 (0.0)	0 (0.0)	2 (7.4)	1 (14.2)
	**Diagnosis of Health Conditions**				**Cervical Manipulation**			
*Full Access*	4 (14.2)	13 (72.2)	17 (62.9)^a^	3 (42.8)	3 (10.7)	12 (66.6)	20 (74.0)	1 (14.2)
*Limited Access*	7 (25.0)	3 (16.6)	5 (18.5)^a^	1 (14.2)	3 (10.7)	3 (16.6)	3 (11.1)	2 (28.5)
*Permitted but NOT in Curriculum*	0 (0.0)	0 (0.0)	0 (0.0)	1 (14.2)	1 (3.5)	0 (0.0)	0 (0.0)	1 (14.2)
*Not Allowed*	17 (60.7)	2 (11.1)	4 (14.8)^a^	2 (28.5)	18 (64.2)	3 (16.6)	4 (14.8)	2 (28.5)
*Unsure*	0 (0.0)	0 (0.0)	0 (0.0)	0 (0.0)	3 (10.7)	0 (0.0)	0 (0.0)	1 (14.2)
	**Taking Blood Samples**				**General Manipulation**			
*Full Access*	0 (0.0)	9 (50.0)	10 (37.0)	1 (14.2)	6 (21.4)	14 (77.7)	22 (81.4)	3 (42.8)
*Limited Access*	5 (17.8)	4 (22.2)	9 (33.3)	2 (28.5)	4 (14.2)	1 (5.5)	2 (7.4)	1 (14.2)
*Permitted but NOT in Curriculum*	2 (7.1)	0 (0.0)	0 (0.0)	2 (28.5)	5 (17.8)	1 (5.5)	0 (0.0)	1 (14.2)
*Not Allowed*	21 (75.0)	5 (27.7)	8 (29.6)	2 (28.5)	12 (42.8)	2 (11.1)	3 (11.1)	1 (14.2)
*Unsure*	0 (0.0)	0 (0.0)	0 (0.0)	0 (0.0)	1 (3.5)	0 (0.0)	0 (0.0)	1 (14.2)
	**Intravenous Administration**				**Naturopathic treatment Prescription**			
*Full Access*	0 (0.0)	9 (50.0)	9 (33.3)	1 (14.2)	21 (75.0)	17 (94.4)	27 (100.0)	6 (85.7)
*Limited Access*	4 (14.2)	4 (22.2)	8 (29.6)	1 (14.2)	3 (10.7)	1 (5.5)	0 (0.0)	1 (14.2)
*Permitted but NOT in Curriculum*	3 (10.7)	0 (0.0)	0 (0.0)	0 (0.0)	0 (0.0)	0 (0.0)	0 (0.0)	0 (0.0)
*Not Allowed*	21 (75.0)	5 (27.7)	10 (37.0)	3 (42.8)	1 (3.5)	0 (0.0)	0 (0.0)	0 (0.0)
*Unsure*	0 (0.0)	0 (0.0)	0 (0.0)	2 (28.5)	1 (3.5)	0 (0.0)	0 (0.0)	0 (0.0)
	**Minor Surgery**				**Sale of Naturopathic Products within Clinic**			
*Full Access*	1 (3.5)	9 (50.0)	10 (37.0)	0 (0.0)	15 (53.5)^a^	13 (72.2)	21 (77.7)	6 (85.7)
*Limited Access*	2 (7.1)	3 (16.6)	4 (14.8)	1 (14.2)	2 (7.1)^a^	1 (5.5)	0 (0.0)	0 (0.0)
*Permitted but NOT in Curriculum*	2 (7.1)	0 (0.0)	0 (0.0)	0 (0.0)	2 (7.1)^a^	0 (0.0)	0 (0.0)	0 (0.0)
*Not Allowed*	23 (82.1)	6 (33.3)	13 (48.1)	6 (85.7)	8 (28.5)^a^	4 (22.2)	4 (14.8)	1 (14.2)
*Unsure*	0 (0.0)	0 (0.0)	0 (0.0)	0 (0.0)	0 (0.0)	0 (0.0)	2 (7.4)	0 (0.0)
	**Pharmaceutical Prescription**				**Non-Conventional Laboratory tests (urine/hair/stool)**			
*Full Access*	0 (0.0)	7 (38.8)	8 (29.6)	0 (0.0)	16 (57.1)	13 (72.2)	20 (74.0)	5 (71.4)
*Limited Access*	3 (10.7)	4 (22.2)	5 (18.5)	1 (14.2)	2 (7.1)	2 (11.1)	5 (18.5)	0 (0.0)
*Permitted but NOT in Curriculum*	1 (3.5)	0 (0.0)	0 (0.0)	0 (0.0)	2 (7.1)	1 (5.5)	0 (0.0)	1 (14.2)
*Not Allowed*	23 (82.1)	6 (33.3)	13 (48.1)	6 (85.7)	7 (25.0)	2 (11.1)	2 (7.4)	0 (0.0)
*Unsure*	0 (0.0)	0 (0.0)	0 (0.0)	0 (0.0)	1 (3.5)	0 (0.0)	0 (0.0)	1 (14.2)

^a^Adjusted for missing data; ^b.^Some respondents reported multiple regulation types; ^c^ Analysis excluded Belgium, Egypt, Hong Kong, State (USA) which did not indicate regulation type, see supplementary information

Sale of naturopathic products directly to clients was more commonly reported where naturopaths were regulated compared to unregulated. (see Table 8). Almost one-quarter of respondents (21.5%) (*n=14*) reported in-clinic sales of naturopathic products not permitted. Prescription for naturopathic treatment (6.2%) (*n=4*) was not allowed in Belgium, Egypt, Florida [USA], and Venezuela (see supplementary information).

Of 65 total respondents, more than half reported some access to general manipulation (66.2%) (*n=43*), followed by acupuncture (55.4%) (*n=36*) with fewer reporting ability to undertake cervical manipulation (47.7%) (*n=31*) (see supplementary information). Access was reported even with regulation of chiropractic, osteopaths, and acupuncturists, indicating shared access to therapies between profession, although potentially with some limitations.

Of the 65 total respondents, ability to write pharmaceutical prescriptions (full access, limited, permitted but not in curriculum) was reported by approximately one-quarter (26.1%, *n*=17) of respondents, most commonly in regulated jurisdictions. A minority of respondents (15.4%) (*n=10*) reported physical examination being restricted, and for almost one third of respondents (32.3%) (*n=21*) laboratory pathology testing was not permitted.

#### Perception of naturopathic regulation

Respondent organizations (95.4%) (*n=*62) commonly reported support for regulation of naturopathy, although opposition to further regulation was reported by Germany, where long-standing ‘*Heilpraktiker’* regulation provides access to a broad range of therapies. The Swedish respondent which reported self-regulation of education and ‘defined scope of practice’ was undecided about statutory regulation due to concerns about more restrictive practice, and the organization from Egypt was unsure.

Statutory regulation was perceived to provide public protection from unqualified or incompetent practitioners and to overcome lack of practice control systems, and diploma mills that provided low quality education, as the following representative quotes indicate:

*“… There are education providers who are offering naturopathic education without government approval to innocent members of the public who have no awareness of the quality of these non-approved programs. With no formal registration of the profession there is no enforceable national standard”* (Australian professional association).

“*As naturopathy is not regulated, and membership to professional organizations is voluntary, there is little control over safety of practitioners once they are qualified and beyond the supervision of our College*” (NZ educational institution).

Regulation was perceived to raise and enforce education and practice standards, provide public safety, state recognition, title protection, public access to reimbursement schemes for costs associated with naturopathic care, and to overcome issues associated with equal employment opportunities in national health systems.

However, regulation of naturopathy was reported to not necessarily provide legitimacy and acceptance by other health profession, nor guarantee state alliance and benefits afforded other health practitioners, as indicated by the following representative quote.

*“We were being penalized by the Medical Association saying that we are not professions of Health and for this reason we had to pay the VAT- tax (tax on value added) … They … put in Tax Enforcement”* (Portugal professional association).

Instruments of occupational control reported by respondents included the establishment of an accreditation board to regulate education, development of national curriculum guidelines, educational consortiums, professional association standards, self-regulation body, and professional codes used to differentiate the qualified from the unqualified, to provide public certainty around naturopathic education, to advance the profession and provide commercial advantage.

In jurisdictions where naturopathy was regulated, respondents reported formalized frameworks that linked regulation with education and entry to profession providing consistent quality control mechanisms for education providers and practitioners entering practice, as the following representative quotes indicate.

“*Regulators accept educational standards set by the CNME in the USA; thus, regulators tend to have standards across North America in respect to examination and licensure*” (Canada professional association).

“*Any education and training in naturopathy requires statutory health council approval against its mandate to protect the health of the public*” (South Africa regulator).

Respondents perceived naturopathic regulation to be politically influenced and circumvented by other health professions protecting market share. Almost one-third of respondents (31%, *n=20*) reported opposition from medical associations, osteopathic medical, and other health professional associations – nurses, physiotherapists, pharmacists, dieticians, psychologists, dentists, and some chiropractic associations. A regional professional association in USA reported:

*“[State] affiliate of the American Medical Association; Chiropractic Association, Nursing Association, Pharmacy Association have all testified against our original licensure as well as more recent efforts to expand our scope of pharmaceutical prescribing rights”* (USA regional professional association).

Other respondents described the challenge of overcoming opposition by state bureaucrats who were perceived to subvert the naturopathic contribution to integrative health care by strategic exclusion from policy development in favor of established health profession, as the following quote demonstrates.

“*The current political context is very challenging. There are very complex class conflicts of interest. For example, we were not cited in the WHO GLOBAL REPORT ON TRADITIONAL AND COMPLEMENTARY MEDICINE 2019. The questionnaire was sent to a department of the government responsible for integrative practices, and the respondent concealed all the information about the bachelor's degree in naturopathy, citing only specializations linked to traditional medicine from other health professions. This attitude generated a conflict, the working group that produced most of the technical documents for the technical area of government responsible for traditional medicine and integrative practices, was predominantly composed by naturopaths. We are currently requesting an erratum of the publication for the WHO*” (Brazil educational institution).

Controversy over regulation was reported by the respondent from Germany. Medical doctors were reported to want increased *Heilpraktiker* education, and some osteopaths and homeopaths were reported to favor development of separate professions within or outside the ‘*Heilpraktiker’* framework.

In addition, some organizations reported intra-professional opposition to regulation. Some respondents (12.3%) (*n=8*) from Australia, Italy, Spain, and USA perceived unlicensed naturopaths, mixed natural medicine associations, and professional associations with no economic interest in regulation, hindered progress on regulation in order to maintain their own interests.

#### Factors perceived to impact regulation of naturopaths

Most respondents reported ‘attitude of decision makers’ (87.7%) (*n=57*); ‘opinion of other health practitioner groups (including medical doctors)’ (76.9%) (*n=50*); ‘regulation or scope of other health practitioners’ (73.8%) (*n=48*); and ‘existing legislation’ (73.8%) (*n=48*) as factors impacting the regulation of naturopaths. Where no regulation existed ‘professional unity’ (or lack thereof) was commonly reported to have most impact on naturopathic regulation (85.7%) (*n=24*).

### Access to public resources and reimbursements for naturopathic services

Most respondents reported ‘full or limited access’ to private health insurance (63.1%) (*n=41*), relatively few respondents reported accesses to government funded public health (13.8%) (*n=9*) and medical integration (21.5%) (*n=14*). Where respondents reported ‘title protection’ access to private health insurance (88.9%) (*n=16*), government funded public health (33.3% (*n=6*) and medical integration (55.5%) (*n=10*) were more commonly reported, (see Table 9).

**Table 9:** Access by naturopaths to private and public resources and reimbursements^c^

	Regulation Type				
	Total (*n*=65)	No Regulation (n=28)^b^	Title protection (n=18)^b^	Defined Practice (n=27)^b^	Other (n=7)^b^
Access to private health insurance	N (%)	N (%)	N (%)	N (%)	N (%)
*Full/Limited Access*	41 (63.1)	14 (50.0)	16 (88.9)	20 (74.0)	7 (100.0)
*Not Allowed/Not Applicable*	23 (35.4)	13 (46.4)	1 (5.5)	6 (22.2)	0 (0.0)
*Unsure*	3 (4.6)	1 (3.5)	1 (5.5)	1 (3.7)	0 (0.0)
Access to government funded public health					
*Full/Limited Access*	9 (13.8)^a^	2 (7.1)^a^	6 (33.3)^a^	8 (29.6)^a^	0 (0.0)
*Not Allowed/Not Applicable*	55 (84.6)	25 (89.2)^a^	11 (61.1)^a^	18 (66.6)^a^	7 (100.0)
*Unsure*	0 (0.0)	0 (0.0)	0 (0.0)	0 (0.0)	0 (0.0)
Access to medical integration (hospital privileges)					
*Full/Limited Access*	14 (21.5)^a^	4 (14.2)^a^	10 (55.5)^a^	11 (40.7)^a^	1 (14.2)
*Not Allowed/Not Applicable*	49 (75.4)	23 (82.1)^a^	7 (38.8)^a^	14 (51.8)^a^	6 (85.7)
*Unsure*	1 (1.5)	0 (0.0)	0 (0.0)	1 (3.7)	0 (0.0)
**Method of payment/cost reimbursement for naturopathic services**					
Direct payment by patient (no reimbursement by third-party)					
*Consultation Costs*	43 (66.2)	13 (46.4)	12 (66.6)	20 (74.0)	4 (57.1)
*Naturopathic Product Prescriptions*	42 (64.6)	13 (46.4)	13 (72.2)	20 (74.0)	5 (71.4)
*In-Office treatments*	38 (58.5)	11 (39.2)	13 (72.2)	20 (74.0)	4 (57.1)
Government funded / public health reimbursement					
*Consultation Costs*	6 (9.2)	0 (0.0)	6 (33.3)	6 (22.2)	0 (0.0)
*Naturopathic Product Prescriptions*	0 (0.0)	0 (0.0)	0 (0.0)	0 (0.0)	0 (0.0)
*In-Office treatments*	7 (10.8)	1 (3.5)	6 (33.3)	5 (18.5)	1 (14.2)
Private health insurance reimbursement					
*Consultation Costs*	36 (55.4)	13 (46.4)	13 (72.2)	19 (70.3)	4 (57.1)
*Naturopathic Product Prescriptions*	14 (21.5)	5 (17.8)	4 (22.2)	4 (14.8)	1 (14.2)
*In-Office treatments*	36 (55.4)	11 (39.2)	14 (77.7)	18 (66.6)	4 (57.1)
Other third-party funder reimbursement (e.g. Veteran’s affairs, Employer-funded workplace injury insurance)					
*Consultation Costs*	10 (15.4)	2 (7.1)	4 (22.2)	6 (22.2)	1 (14.2)
*Naturopathic Product Prescriptions*	1 (1.5)	0 (0.0)	0 (0.0)	0 (0.0)	0 (0.0)
*In-Office treatments*	7 (10.8)	1 (3.5)	3 (16.6)	4 (14.8)	1 (14.2)
Not applicable					
*Consultation Costs*	10 (15.4)	9 (32.1)	0 (0.0)	1 (3.7)	0 (0.0)
*Naturopathic Product Prescriptions*	10 (15.4)	8 (28.5)	0 (0.0)	1 (3.7)	0 (0.0)
*In-Office treatments*	9 (13.8)	7 (25.0)	0 (0.0)	1 (3.7)	0 (0.0)
**Factors that impact patient cost reimbursement of naturopathic services**					
*Practitioner must have malpractice insurance*	21 (32.3)	11 (39.2)	5 (27.7)	11 (40.7)	3 (42.8)
*Practitioner must have registered title/number from regulatory body*	30 (46.2)	9 (32.1)	11 (61.1)	18 (66.6)	4 (57.1)
*Patient characteristics (*e.g. *veterans, workplace, injury, elderly)*	3 (4.6)	1 (3.5)	3 (16.6)	3 (11.1)	0 (0.0)
*Diagnosed disease/condition*	13 (20.0)	2 (7.1)	8 (44.4)	9 (33.3)	2 (28.5)
*Level of practitioner education/qualification*	19 (29.2)	10 (35.7)	7 (38.8)	9 (33.3)	2 (28.5)
*Referral from a medical practitioner*	8 (12.3)	3 (10.7)	3 (16.6)	3 (11.1)	1 (14.2)
*Type of therapies /treatments provided by the practitioner*	24 (36.9)	7 (25.0)	11 (61.1)	14 (51.8)	3 (42.8)
*Other*	13 (20.0)	6 (21.4)	5 (27.7)	7 (25.9)	1 (14.2)
*Not applicable*	19 (29.2)	12 (42.8)	1 (5.5)	3 (11.1)	1 (14.2)

^a^Adjusted for missing data; ^b.^Some respondents reported multiple regulation types.^c^ Analysis excluded Belgium, Egypt, Hong Kong, State (USA) which did not indicate regulation type

Most respondents reported the most common payment options for naturopathic services were direct out of pocket followed by private health insurance. Almost two thirds of respondents (66.2%) (*n=43*) reported out of pocket payment for naturopathic consultations, product prescriptions (64.6%) (*n=42*), and in-office treatments (58.5%) (*n=38*). Respondents reported some reimbursement by private health insurance for consultations 55.4% (*n=36*), product prescriptions (21.5%) (*n=14*) and in-office treatments (55.4%) (*n=36*) (see Table 9), with coverage of consultation costs and in-office treatments more frequently reported when the profession was regulated.

Consumer access to reimbursement of costs for naturopathic services where unregulated, were reported to be dependent on practitioner holding malpractice insurance (39.2%) (*n=11*), or the practitioner’s level of education and qualification (35.7%) (*n=10*), or a registration title or number from a voluntary regulatory board (32.1%) (*n=9*). Reimbursements where naturopathic medicine was regulated were reported to be dependent on practitioner holding a registered title or number issued by regulatory board, type of therapies or treatment provided (‘title protection’ 61.1%, [*n=11*]) (‘defined scope of practice’ 51.8%, [*n=14*]), diagnosed disease or condition (‘title protection’ 44.4%, [*n=8*]) and practitioner holding malpractice insurance (‘defined scope of practice’ 40.7%, [*n=11*]) (see Table 9). Using a seven-point rating scale, most respondents (56.9%) (*n=37*) perceived reimbursement of costs associated with naturopathic care to be ‘more than moderately’ tied to regulation (M=6), integration (55.3%) (*n=36*)] (M=6) and utilization of naturopathic services (56.9%) (*n=37*) (M=6).

## Discussion

The results presented here represent the first in-depth examination of global naturopathic regulation, education, and policy frameworks that constrain professionalization and integration into health systems, as perceived by the naturopathic community. Key findings serve to inform policy development by state, professional bodies, educational institutions, and third-party funders for development of frameworks that support professional formation, public access, and integration into national health systems. Closer examination of the interface between country, regulation, and education frameworks should be considered in future research.

### Regulation

#### Diverse regulation is a barrier to consistent education, professional frameworks, and integration

Our study demonstrated significant heterogeneity and wide-ranging methods of global naturopathic regulation, which may act as a barrier to development of consistent global educational and professional frameworks, and integration. This may in part be compounded by the difficulties in many countries to accurately capture naturopathic medicine in legislation. Broad terminology (e.g. ‘natural medicine’) or more narrow definitions based on specific therapeutic tools (e.g. ‘herbal medicine’) may be used by governments, as this is often easier to practically implement than regulation of a complex system of traditional medicine. This would explain variations within the WHO 2019 T&CM report, which reported population use of naturopathy in 98 of 133 countries (73.6%), yet practicing naturopaths in 35 countries (26.3%) [25], and differences with WNF reported presence of naturopathic practitioners in 81 countries [9].

The recommendation by the WHO that national policy development of T&CM practitioners and practices is the responsibility of member states [26], without provision for clear guidance, potentially underpins this issue. An alternative explanation for the results, is habituated denial of naturopathic practitioners’ contribution to healthcare, an exclusionary tactic reported by Brazil in our study, but one that has also been reported by other professions [27].

Inconsistent or delayed regulation of T&CM professions may potentially be less about risk management – control of sub-standard education, practice standards, and public safety, and more about national politics and established state-profession relations – and may reflect professional tensions between biomedicine and complementary medicine, as reported elsewhere [21, 28–30]. Prejudice against professionalization of T&CM practitioners by established health profession undertaking boundary construction, jurisdictional protection, and expansion of practices, supported by enduring structural arrangements, is well documented in the literature in many of the countries that have been part of this study. In South Africa, for example, the *Medical Association of South Africa* in 1953 declared naturopathy unscientific, and through code of ethics, prohibited collaboration between doctors and naturopathic practitioners, which has been reinforced through regulatory Acts [21]. Inconsistent and delayed regulation by the state enables continued biomedical hegemony and economic self-interest, marginalization of naturopathic practitioners and consumers, and negates broader interests of society.

Our results confirm and extend findings from the pan-European CAMbrella project [19, 23], which found EU regulation directing member states to develop T&CM health policy, resulted in diverse regulation that was non-conformant with *EU Cross-border Healthcare Directive* and out of step with current theory of risk management, risk regulation and patient safety. In this study, patient’s seeking T&CM across borders encountered different training of identical T&CM practitioners, and different reimbursement systems. Diverse regulation was found to be a barrier to consistent delivery of T&CM treatment and research [23, 31]. Our study suggests heterogeneity extends to include inconsistent global delivery of education, development of professional frameworks, and integration.

### Integration of naturopathic medicine into health care systems

We found limited reporting of integration of naturopathic services in healthcare systems in 29 countries, although there are other examples of state-funded naturopathic services. In India, the separate Ministry of AYUSH (abbreviation for Ayurveda, Yoga and Naturopathy, Unani, Siddha and Homeopathy) is responsible for mainstreaming this range of T&CM medical systems, and in 2014, forty-two AYUSH hospitals providing 1107 beds, were reported to provide yoga and naturopathy services [32].

In our study, access to private health insurance, government funded public health and medical integration were more frequently reported where ‘title protection’ forms of regulation existed. Full access to government funded public health, was only reported in USA (and then only in some States), and full medical integration only reported in Congo DR, Zambia, and Switzerland. Lack of integration may create additional difficulties for patients who wish to use naturopathy in addition to other services. Individuals with chronic health conditions use T&CM care regularly and frequently for condition management, and limited system interface may mean consumers have to manage and negotiate parallel systems, determining interpretation, and sharing of health information between healthcare providers [33].

Limited medical integration of naturopathic medicine reported in our study is consistent with findings in chiropractic, where chiropractic integration was limited to several hospital outpatient settings [34]. Integration of naturopathy into health systems requires political will, potentially driven by a demanding public, as demonstrated by Switzerland constitutional referendum in 2009 that resulted in inclusion of complementary medicine in *Article 118a of the Switzerland Constitution* [35] and in 2017, inclusion of some T&CM in mandatory health insurance when delivered by dual trained conventional doctors [36].

Consistent with our findings, the last major Australian (Victorian) government review into the regulatory and legislative requirements for naturopathy, found lack of direct legislation counter to development of consistent standards of education and professional standards, resulting in difficulties in enforcing and sustaining minimum standards of training and practice [37]. We argue diverse regulation, and subsequent variation in education and professional frameworks, may not support WHO T&CM (2014–2023) strategic objectives to: build a knowledge base to support evidence-based practice; strengthen safety, quality and effectiveness; and promote universal health coverage [26]. We propose that growth in naturopathic educational programs in the past 25 years, in an environment of limited or inconsistent regulation, with absence of consistent frameworks and minimal WHO training guidelines, has fostered differences in global naturopathic education and hindered professional formation. Moreover, we propose that although naturopathic practice is impacted by varying regulatory dynamics and structural and cultural differences, consistency in education frameworks operationalized within and between countries, will assist professional formation, integration, practitioner transportability and user access.

### Education and practice standards

#### Primary contact care demands higher education training

In our study, global naturopathic training varied between two to four years in length, with a presence primarily in the higher education sector, although vocational qualifications were reported by educational institutions from Belgium, Czech Republic, France, Italy, Nepal, Slovenia, Uruguay, and Venezuela. Almost two-thirds of educational institutions, mostly private, reported delivery of bachelor’s or postgraduate qualifications via a national qualifications’ framework with almost half delivering four-year programs. A previous study by McCabe found that the degree-level of training is the minimum preparation required for independent, primary contact practice in naturopathy, which requires a culture of enquiry, interaction with other health professionals, and understanding of practice limitations [12]. This is consistent with other T&CM professions providing primary-point-of-care practice such as chiropractic [20], and primary care nurse practitioners in private practice [38]. On this basis the WHO naturopathic training guidelines should be considered only the minimum requirement for entry-level technician roles [1].

#### Voluntary organizations lack legal mandate, have potentially inadequate regulatory frameworks, and conflicting interests

Educational institutions in our study were reported to have significant influence on faculty characteristics and programs, with accreditation bodies influential where there was regulation, and government departments and professional associations where programs were delivered on a national qualifications’ framework. Where naturopathic education was delivered outside these parameters, professional associations provided limited oversight based on organizational standards. However, voluntary professional associations may not be equipped for this role, and studies in Australia and the United Kingdom have shown them to be insufficient in this role as it relates to naturopathy [37, 39]. Attempts to complement professional associations with voluntary registers has also been largely ineffective [40, 41].

In our study, presence of unified national representation of the naturopathic profession was not ubiquitous, and multiple competing national and mixed associations – with differing views on standards and professional priorities – were a reported obstacle to advancement of naturopathic education and the naturopathic profession. Professional unity (or lack thereof) was perceived to impact regulation in some unregulated jurisdictions. This finding supports previous qualitative examination of Australian naturopaths where factional agendas and conflicts within competing associations was found to delay registration, higher education standards, as well as other professional development activities [42]. Intra-professional conflict has been a previously reported barrier to professionalization in Portugal, where lack of cohesion and infighting between T&CM groups delayed regulation of naturopathy and other disciplines by 10 years [19] and in Ontario, where only those T&CM groups with good internal communication, organizational infrastructures, and fit with the healthcare system, achieved regulation [43].

#### Development of global education standards and accreditation agencies a priority

In our study naturopathic programs were primarily based on local education standards. We found limited evidence, other than CNME accredited programs in North America, of consistent education frameworks and attempts for harmonization of programs across countries. This is in stark contrast to other T&CM professions such as chiropractic which has harmonized education frameworks across world regions [44]. Chiropractic established an international umbrella council, *Council on Chiropractic International* (CCE-International) with representatives from four regional *Councils on Chiropractic Education* (CCE) [Australasia, Canada, Europe and USA] all contributing to development of the *Accreditation Standards for the CCE-International* [44]. CCE-International is the body recognized by WHO as the source for evaluation of chiropractic education, and guides education development globally with accreditation of educational programs undertaken by four regional accreditation bodies [44].

The naturopathic profession has not developed an equivalent body, but the development of the *World Naturopathic Federation* may provide an avenue for organizations such as WHO, to provide a basis for development of global standards potentially for different forms of practice. Existing regional standards and competencies for the spectrum of regulated naturopathic disciplines - naturopathic doctor, naturopath and naturopath technician [9], could form the basis of international standards and competencies, indirectly supporting national regulation and naturopathic integration into health systems. Our study indicated that much of this work is quite advanced at a national level in Australia, Brazil, Canada, Switzerland, and the United States, which could provide a basis for development of global naturopathic standards and competencies for alignment by world regions.

## Limitations

Findings from this study should be viewed within the context of its limitations. Due to the nature of the survey, and overlapping regulatory groups, analysis is largely descriptive. As the survey was limited to the English language there may have been some cultural or linguistic differences in interpretation, reporting and participation. Additionally, not every country was represented as educational institution participation was limited to 16 of 25 countries, professional associations to 25 of 44 countries, and regulatory boards to 5 of 7 countries. Some countries were not represented by both educational institutions and professional associations, limiting ability to examine education frameworks and accountability mechanisms relative to professional frameworks. The extended timeframe of this survey and the evolving nature of jurisdiction may mean data collected early may not be directly comparable with data collected near the end of the project. This study was also limited to organizations self-reporting and did not examine policy, nor organizational documents for concordance between what was described and praxis, all of which may warrant further more detailed examination. Nevertheless, the results of this study form the most comprehensive global examination of the education, regulatory landscape of naturopathic practice to date, and as such offer valuable insights to the naturopathic profession, policy-makers, and other key professional stakeholder groups.

## Conclusion

Education and regulation of the naturopathic profession has significant heterogeneity, even in the face of global calls for consistent regulation that recognizes naturopathy as a medical system. Standards are highest and consistency more apparent in countries with regulatory frameworks. Consistent regulation, formalized education frameworks and practice standards, could potentially support increased professional formation, integration of naturopaths into health systems and increased access to naturopathic care by the public.

## Supplementary Information

**Additional file 1.**

**Additional file 2.**

**Additional file 3.**

## Data Availability

Survey instrument attached. The dataset generated during the current study are not publicly available due to potentially identifying information but is available from the corresponding author on reasonable request.

## Abbreviations

CCE: Councils on Chiropractic Education
CCE-International: Council on Chiropractic International
CNME: Council on Naturopathic Medical Education
ISO: International Standards Organization
NPLEX: Naturopathic Profession Licensing Examination
T&CM: Traditional and Complementary Medicine
UNI: Ente Nazionale Italiano di Unificazione
WHO: World Health Organization
WNF: World Naturopathic Federation
