# Supplementary material for: Characteristics of global naturopathic education, regulation, and practice frameworks: results from an international survey
Source: BMC Complement Med Ther. 2021 Feb 18;21:67. doi: 10.1186/s12906-021-03217-1 (PMC7893718; doi:10.1186/s12906-021-03217-1)
Supplement: Supplementary file 1 — Additional file 1. [file 12906_2021_3217_MOESM1_ESM.pdf]

WORLD NATUROPATHIC FEDERATION

Mapping the regulatory and educational landscape for naturopathy

Information Sheet

ID 1126

Who is doing the research?

My name is Jill Dunn and I am a naturopath, academic leader at Wellpark College of Natural Therapies, New Zealand and PhD student in the Faculty of Health at the University of Technology Sydney (UTS), Australia. I am also chair of the World Naturopathic Federation (WNF) Professional Mapping Committee. My supervisors are Dr Jon Wardle, Chancellor's Research Fellow at the Faculty of Nursing Midwifery and Health and Acting Co-Secretary General of World Naturopathic Federation; Dr Amie Steel, Post-doctoral Research Fellow UTS, Chair of the Australian Register of Naturopaths and Herbalists and Professor Jon Adams, Professor of Public Health, ARC Professional Future Fellow and Director of the Australian Research Centre in Complementary and Integrative Medicine [ARCCIM] at the Faculty of Health at UTS.

Data collection, survey development and publishing of results is being carried out in collaboration with WNF, namely: Dominick Léaud-Zachoval (Director of Education, Aesculape School of Naturopathy and President of FENAHMAN, France; Tina Hausser (Vice-President for International Affairs of the Spanish Association of Naturopaths "Organización Colegial Naturopática OCN FENACO" and Vice-President of the World Naturopathic Federation), Spain; Professor Dr Karki (Secretary Nepal Yoga and Nature Cure Association, Chairman of both Institute of Natural Medicine and Nature Cure Research and Teaching Hospital) Nepal, Dr Nicholas deGroot (Dean of the Canadian College of Natural Medicine, Canada), Dr Iva Lloyd (President of the World Naturopathic Federation).

ID 1127

What is this research about?

The purpose of this research /online survey is to examine the relationship between naturopathic education and regulation and to what degree they influence each other and support or hinder global professional development of naturopathy. We will examine global influences and the part that regulation and professional associations play in naturopathic education, standards and competencies.

Following on from the survey, I will request some organisational documents that may or may not be in the public domain, in order to examine how policies, frameworks and infrastructures globally impact on standards, curriculum and scope of practice. All documents whether in the public domain or not will be treated as confidential. Documents will only be accessed by the researcher and supervisors and a translator from WNF where required. Translators will be required to sign a confidentiality agreement. These documents will not be provided to other individuals, government departments or any other organisation. Any information used from these documents will be de-identified or generalised in the final report so that sources of the original documents and data will not be disclosed. Documents will be destroyed at the participant's request, or archived with completed questionnaires in an electronic password protected confidential folder, backed up regularly for seven years following completion of this research qualification.

Findings from the survey and document analysis will be disseminated to the naturopathic profession through a report from World Naturopathic Federation, conference presentations and published articles in peer-reviewed journals, to assist in decision making.

Information Sheet continued

ID 1128

If I say 'Yes', what will it involve?

As an office-bearer of your professional organisation, we will ask you to complete an online questionnaire that may take up to 60 minutes to complete. You are required to have the organisational authority to complete this survey. Your completion of this survey will be taken as your organisations and your consent to participate. The completion date for this survey is April 30th, 2017.

ID 1129

Are there any risks or inconvenience?
Yes there are some risks and inconveniences. This survey will take up to 60 minutes to complete.

This survey is not anonymous although confidential; participants and professional organisations will be de-identified. Office-bearer contact details will be stored in a password protected electronic file as some participants may be invited to participate in a semi-structured interview in the future as part of this research project.

This study involves examination of organisational practices and there is likely to be variance in the stage that the profession is at globally. Competing professional associations and education establishments are likely to take part in this survey, and therefore some answers may be considered “sensitive”. To avoid identification all data will be de-identified and comparisons will be generalised.

This survey will be provided in English; if English is not your native tongue there is a risk of misinterpretation of the questions and we would request that you seek assistance from another member of your organisation for the purpose of translation. Office-bearers who complete the survey will be de-identified in this study.

If you feel uncomfortable with a question you can skip that question or withdraw from the survey altogether. You can change your mind at any time and stop completing the survey without consequences.

ID 1170

Why have I been asked?

You have received this invitation to participate in this global online survey because you are part of an organisation/institution that regulates naturopaths (as part of its role) or because your organisation/institution represents the profession of naturopaths or educates naturopathic practitioners. You have been identified as a stakeholder by WNF as your organisation/institution is believed to regulate, represent or educate practitioners who:

1. Are primarily trained as naturopaths

2. Have met or exceeded the World Health Organisation benchmark for education and training of:
• a minimum of two years
• no less than 1500 hours
• a minimum of 400 hours clinical training (is included unless country standards differ).

3. Have gained their naturopathic qualification through attendance or both attendance and online/distance education.

ID 1131

Do I have to say 'Yes'?
You don’t have to say yes. Your participation is completely voluntary.

What will happen if I say 'No'?
Nothing. We will thank you for your time so far and won’t contact you about this research again.

If I say 'Yes', can I change my mind later?
You can change your mind at any time and you don’t have to say why. We will thank you for your time so far and won’t contact you about the research again.

Information Sheet continued

ID 1133

What if I have concerns or a complaint?
If you have concerns about the research that you think I or my supervisors can help you with, please feel free to contact us:

Jill Dunn
Wellpark College of Natural Therapies
PO Box 78 229
Grey Lynn
Auckland 1245
New Zealand

Email: Jillian.M.Dunn@student.uts.edu.au
Skype: jill.dunn10

Phone: +64 9 360 0560

or my supervisors:

Dr Jon Wardle (Chancellor's Research Fellow at the Faculty of Nursing Midwifery and Health, University of Technology Sydney); Acting Co-Secretary General World Naturopathic Federation.
Email: jon.wardle@uts.edu.au
Phone: +61 2 9514 4813

Dr Amie Steel (Post-doctoral Research Fellow UTS, Associate-Director of Research at Endeavour College of Natural Health/Chair of the Australian Register of Naturopaths and Herbalists, Australia)
Email: amie.steel@uts.edu.au
Phone: +61 7 3253 9523

Professor Jon Adams (Professor of Public Health, ARC Professorial Future Fellow and Director of the Australian Research Centre in Complementary and Integrative Medicine (ARCCIM) at the Faculty of Health at UTS).
Email: jon.adams@uts.edu.au

Phone: +61 2 9514 4821

If you would like to talk to someone who is not connected with the research, you may contact the Research Ethics Officer on 02 9514 9772 or research.ethics@uts.edu.au and quote this number **ETH-16-0865**.

ID 1134

Who is my local contact for this survey?
Contact details for your local World Naturopathic Federation representative can be found on the WNF website here

Or:

Dominick Léaud-Zachoval (France) [Europe contact except Spain and Portugal]
Email: dlznaturo@orange.fr

Niloofar Tochaie (Spain) (Europe contact Spain and Portugal]
Email: niloofar_tochaie@yahoo.com

Professor Dr Karki (Nepal) (S.E. Asia contact)
Email: info@naturallifes.com

Dr Nick deGroot (Canada)
Email: NDegroot@ccnm.edu

If you would like to contact World Naturopathic Federation president about this research, please contact Dr Iva Lloyd
Email: president@worldnaturopathicfederation.org

Consent Form

ID 1135

If you agree to be part of this research project and for data gathered from this survey to be published in a form that does not identify you personally, please continue with answering the survey questions.

Please be aware that this survey will take approximately 30-60 minutes to complete. You will be able to save a partially completed survey and return at a later time to complete your responses by selecting the link at the top of your screen. Please, however, ensure you have adequate time to complete the survey before starting.

ID 1212

1. Click the 'Save and Continue later' link at the top of your screen

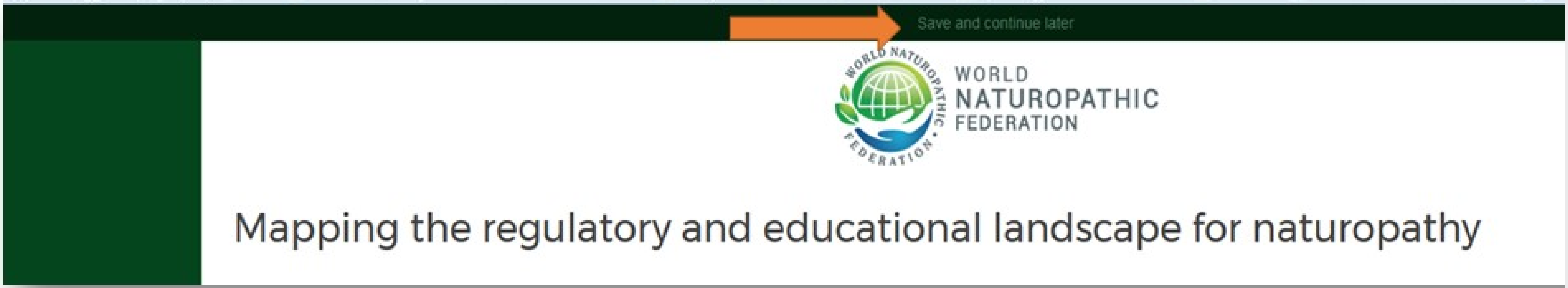

2. Enter your email address

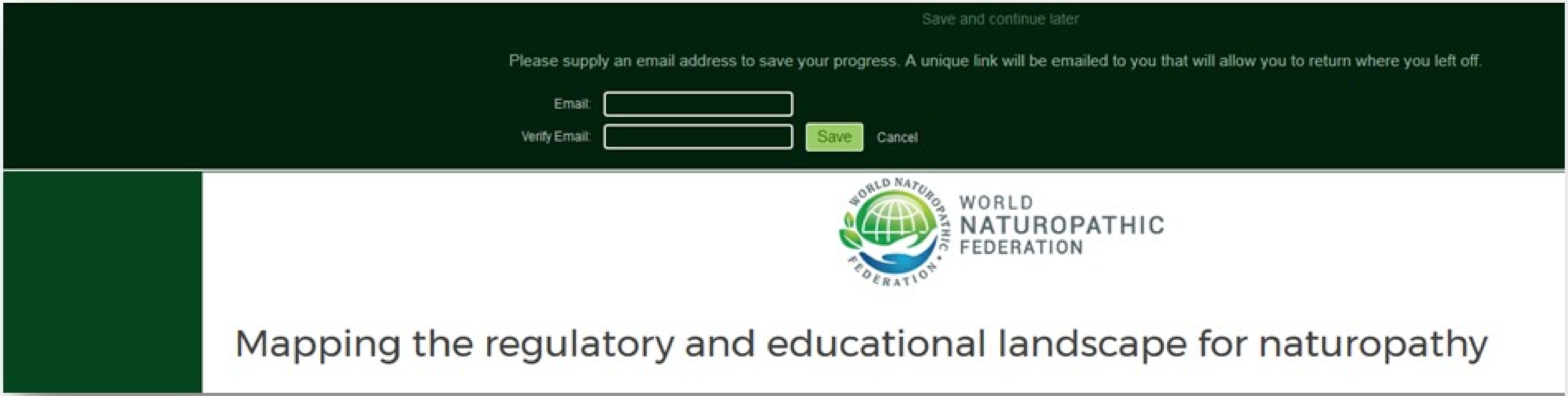

3. Store the link you receive in your email inbox in a safe location until you are ready to complet
4. Click the link in your email and you will be redirected to your incomplete survey

Eligibility

Page exit logic: Skip / Disqualify Logic
IF: Question "Which of the following best describes your organisation?" #1 is one of the following answers ("None of the above") **THEN:** Jump to page 29 - Thank You! Flag response as complete

LOGIC Show/hide trigger exists.
ID 5
1. Which of the following best describes your organisation?

- ○ Educational institution with a specific program that trains naturopathic practitioners
- ○ Naturopathic professional association
- ○ Organisation that regulates the practice of naturopathy
- ○ None of the above

ID 6
2. Which of the following criteria describes the naturopathic training/education programme delivered or supported by your institution? Please select ALL that apply.

- ☐ At least 1500 contact/teaching hours (or equivalent)
- ☐ Minimum of 2 years in length
- ☐ Minimum of 400 hours of supervised clinical practicum in a residential clinic setting
- ☐ None of the above

Education institution/Organisational Contact Details

Page description:
These contact details are de-identified in extracted survey data. They are kept for the purpose of follow up research as part of this research project and to email you a completed report of this research project. Contact details will not be shared with any individual, institution, organisation or government department.

ID 12

3. What is the name of your education institution/organisation?

ID 13

4. What country do you reside in?

- Option 1
- Option 2
- Afghanistan
- Albania
- Algeria
- Andorra
- Angola
- Antigua and Barbuda
- Argentina
- Armenia
- Australia
- Austria
- Azerbaijan
- Bahamas, The
- Bahrain
- Bangladesh

Bangladesh
Barbados
Belarus
Belgium
Belize
Benin
Bhutan
Bolivia
Bosnia and Herzegovina
Botswana
Brazil
Brunei
Bulgaria
Burkina Faso
Burundi
Cambodia
Cameroon
Canada
Cape Verde
Central African Republic
Chad
Chile
China
Colombia
Comoros
Congo, Democratic Republic of the
Congo, Republic of the
Costa Rica
Cote d'Ivoire
Croatia
Cuba
Curacao
Cyprus
Czech Republic
Denmark
Djibouti
Dominica

Dominican Republic
East Timor (see Timor-Leste)
Ecuador
Egypt
El Salvador
Equatorial Guinea
Eritrea
Estonia
Ethiopia
Fiji
Finland
France
Gabon
Gambia, The
Georgia
Germany
Ghana
Greece
Grenada
Guatemala
Guinea
Guinea-Bissau
Guyana
Haiti
Holy See
Honduras
Hong Kong
Hungary
Iceland
India
Indonesia
Iran
Iraq
Ireland
Israel
Italy

Jamaica
Japan
Jordan
Kazakhstan
Kenya
Kiribati
Kosovo
Kuwait
Kyrgyzstan
Laos
Latvia
Lebanon
Lesotho
Liberia
Libya
Liechtenstein
Lithuania
Luxembourg
Macau
Macedonia
Madagascar
Malawi
Malaysia
Maldives
Mali
Malta
Marshall Islands
Mauritania
Mauritius
Mexico
Micronesia
Moldova
Monaco
Mongolia
Montenegro
Morocco

Mozambique
Myanmar
Namibia
Nauru
Nepal
Netherlands
Netherlands Antilles
New Zealand
Nicaragua
Niger
Nigeria
North Korea
Norway
Oman
Pakistan
Palau
Palestinian Territories
Panama
Papua New Guinea
Paraguay
Peru
Philippines
Poland
Portugal
Qatar
Romania
Russia
Rwanda
Saint Kitts and Nevis
Saint Lucia
Saint Vincent and the Grenadines
Samoa
San Marino
Sao Tome and Principe
Saudi Arabia
Senegal
[illegible]

Serbia
Seychelles
Sierra Leone
Singapore
Slovakia
Slovenia
Solomon Islands
Somalia
South Africa
South Korea
South Sudan
Spain
Sri Lanka
Sudan
Suriname
Swaziland
Sweden
Switzerland
Syria
Taiwan
Tajikistan
Tanzania
Thailand
Timor-Leste
Togo
Tonga
Trinidad and Tobago
Tunisia
Turkey
Turkmenistan
Tuvalu
Uganda
Ukraine
United Arab Emirates
United Kingdom
United States
Uruguay

Uruguay
Uzbekistan
Vanuatu
Venezuela
Vietnam
Yemen
Zambia
Zimbabwe

LOGIC Hidden unless: Question "Which of the following best describes your organisation?" #1 is one of the following answers ("Naturopathic professional association","Organisation that regulates the practice of naturopathy")

ID 1074

5. What region does your organisation represent or oversee? (e.g. national, state/province - if not national please specify the region)

- Regional
- National
- Other - Please specify (Required)

*

ID 23

6. Please provide the following details for the most appropriate contact person at your institution/organisation with regards to naturopathy:

First Name

Last Name

Title (Dr, Mr, Ms, Mrs, Miss)

Position/role (e.g. academic leader, president)

Year started in current position

Option 1
Option 2
1950
1951
1952
1953
1954
1955
1956
1957
1958
1959
1960
1961
1962
1963
1964
1965
1966
1967
1968
1969
1970
1971
1972
1973
1974
1975
[illegible]

1976
1977
1978
1979
1980
1981
1982
1983
1984
1985
1986
1987
1988
1989
1990
1991
1992
1993
1994
1995
1996
1997
1998
1999
2000
2001
2002
2003
2004
2005
2006
2007
2008
2009
2010
2011
2012

2012
2013
2014
2015
2016
2017
2018

Street Address

Apt/Suite/Office

City

State

Zip

Country

Afghanistan
Albania
Algeria
Andorra
Angola
Antigua and Barbuda
Argentina
Armenia
Australia
Austria
Azerbaijan
Bahamas, The
Bahrain
Bangladesh

[illegible]
Barbados
Belarus
Belgium
Belize
Benin
Bhutan
Bolivia
Bosnia and Herzegovina
Botswana
Brazil
Brunei
Bulgaria
Burkina Faso
Burundi
Cambodia
Cameroon
Canada
Cape Verde
Central African Republic
Chad
Chile
China
Colombia
Comoros
Congo, Democratic Republic of the
Congo, Republic of the
Costa Rica
Cote d'Ivoire
Croatia
Cuba
Curacao
Cyprus
Czech Republic
Denmark
Djibouti
Dominica

Dominican Republic
East Timor (see Timor-Leste)
Ecuador
Egypt
El Salvador
Equatorial Guinea
Eritrea
Estonia
Ethiopia
Fiji
Finland
France
Gabon
Gambia, The
Georgia
Germany
Ghana
Greece
Grenada
Guatemala
Guinea
Guinea-Bissau
Guyana
Haiti
Holy See
Honduras
Hong Kong
Hungary
Iceland
India
Indonesia
Iran
Iraq
Ireland
Israel
Italy

Jamaica
Japan
Jordan
Kazakhstan
Kenya
Kiribati
Kosovo
Kuwait
Kyrgyzstan
Laos
Latvia
Lebanon
Lesotho
Liberia
Libya
Liechtenstein
Lithuania
Luxembourg
Macau
Macedonia
Madagascar
Malawi
Malaysia
Maldives
Mali
Malta
Marshall Islands
Mauritania
Mauritius
Mexico
Micronesia
Moldova
Monaco
Mongolia
Montenegro
Morocco
[illegible]

Mozambique
Myanmar
Namibia
Nauru
Nepal
Netherlands
Netherlands Antilles
New Zealand
Nicaragua
Niger
Nigeria
North Korea
Norway
Oman
Pakistan
Palau
Palestinian Territories
Panama
Papua New Guinea
Paraguay
Peru
Philippines
Poland
Portugal
Qatar
Romania
Russia
Rwanda
Saint Kitts and Nevis
Saint Lucia
Saint Vincent and the Grenadines
Samoa
San Marino
Sao Tome and Principe
Saudi Arabia
Senegal
Serbia

Serbia
Seychelles
Sierra Leone
Singapore
Slovakia
Slovenia
Solomon Islands
Somalia
South Africa
South Korea
South Sudan
Spain
Sri Lanka
Sudan
Suriname
Swaziland
Sweden
Switzerland
Syria
Taiwan
Tajikistan
Tanzania
Thailand
Timor-Leste
Togo
Tonga
Trinidad and Tobago
Tunisia
Turkey
Turkmenistan
Tuvalu
Uganda
Ukraine
United Arab Emirates
United Kingdom
United States
Uruguay

Uzbekistan
Vanuatu
Venezuela
Vietnam
Yemen
Zambia
Zimbabwe

Email Address

Phone Number

Fax Number

Mobile Phone

Website address

Is the postal address for your institution different to the address provided above?

- [ ] Yes
- [ ] No

LOGIC Hidden unless: Question "Is the postal address for your institution different to the address provided above?" is one of the following answers ("Yes")

ID 238

7. What is the main postal address for your institution?

Company Name

Postal Address

City

State

Zip

Country

New Send Email

To: Amie Steel (research@endeavour.edu.au)

From: SurveyGizmo (noreply@surveygizmo.com)

Subject: New Response Notification

Key terms

ID 1120

For the purposes of this survey, we use the following definitions for key terms:

Region: *The area for which your organisation operates or has jurisdictional influence. For example, a region would be a state or province if that is the focus of your organisation, or encompass an entire country if your organisation has a national scope.*

Naturopath/Naturopathic doctors: *These two terms are used interchangeably throughout the survey to refer to health professionals who have completed formal naturopathic training.*

Clinical supervisor: *A qualified naturopath and academic who provides oversight and guidance to student practitioners providing care to patients from the community as part of structured clinical training within a naturopathic education program*

Naturopathic education program: *A full course of study which results in the award of a qualification which enables the practice of naturopathy in a given region.*

Protection of title: *a legislative approach which restricts individuals from using a title from a particular professional group (e.g. naturopath) without approval by a designated registering body or agency.*

Controlled acts: *acts which may only be performed by authorized regulated health professionals.*

Scope of practice: *the procedures, actions, and processes that a healthcare practitioner is permitted to undertake in keeping with the terms of their professional license.*

Consultation: *an interaction between a patient and a naturopath through which the patient seeks the expert advice of the naturopath*

Treatment: *naturopathic care given to a patient by a naturopath for an illness or injury*

Regulation: *In this instance, regulation refers to statutory regulation which is regulation of the profession through government statute in line with other government regulated professions.*

Naturopathic professional and regulatory environment

ID 1198

8. Which of the following categories of naturopathic organisations exist in your country?

National naturopathic association(s)	none<br>1<br>2<br>3<br>4<br>5<br>6<br>7<br>8<br>9<br>10 or more
Regional naturopathic association(s)	none<br>1<br>2<br>3<br>4<br>5<br>6<br>7<br>8<br>9<br>10 or more
Naturopathic educational institution(s)	none<br>1<br>2<br>3<br>4<br>5<br>6<br>7<br>8

9
10 or more

Naturopathic regulatory body(s)

none
1
2
3
4
5
6
7
8
9
10 or more

Other naturopathic organisation(s)

none
1
2
3
4
5
6
7
8
9
10 or more

Characteristics of educational institution

Page entry logic:
This page will show when: Question "Which of the following best describes your organisation?" #1 is one of the following answers ("Educational institution with a specific program that trains naturopathic practitioners")

ID 167

9. How is your educational institution established?

- [ ] Corporation under a Corporations Act
- [ ] Incorporated partnership
- [ ] Unincorporated partnership
- [ ] Incorporated Joint Venture
- [ ] Unincorporated Joint Venture
- [ ] Incorporated Association
- [ ] Unincorporated Association
- [ ] Trust
- [ ] Co-operative
- [ ] Not for Profit Organisation
- [ ] Statutory Body
- [ ] Other - Write In

ID 168

10. Within the educational institution is there a faculty, school or department specifically responsible for offering the course(s) in naturopathy?

- Yes - provide details
- No

VALIDATION Must be numeric Whole numbers only Positive numbers only

ID 175

11. What was the first year the school ever offered a course in naturopathy?

Please insert a year (e.g. 1970)

Qualifications offered at your institution

Page entry logic:
This page will show when: Question "Which of the following best describes your organisation?" #1 is one of the following answers ("Educational institution with a specific program that trains naturopathic practitioners")

ID 1207

12. Please provide some basic details about each naturopathic program offered at your institution

	What is the title of the course?	What is the level of enrolment for the course?		What is the duration of the course for full time students (in years)?
		undergraduate	postgraduate	
Qualification 1				<1<br>1<br>2<br>3<br>4<br>5<br>6+
Qualification 2				<1<br>1<br>2<br>3<br>4<br>5<br>6+
Qualification 3				<1<br>1<br>2<br>3<br>4<br>5<br>6+

Page entry logic:
This page will show when: Question "Which of the following best describes your organisation?" #1 is one of the following answers ("Educational institution with a specific program that trains naturopathic practitioners")

LOGIC Show/hide trigger exists.
ID 730
13. Does your educational program meet government standards, guidelines or requirements for programs of this kind in your region?

- Yes
- No
- Unsure

LOGIC Hidden unless: Question "Does your educational program meet government standards, guidelines or requirements for programs of this kind in your region?" #13 is one of the following answers ("Yes")
ID 731
14. Please provide the name of the framework used to guide qualification/course development?

LOGIC Show/hide trigger exists.

ID 733

15. Is your educational program approved by an external organisation?

- Yes
- No
- Unsure

LOGIC Hidden unless: Question "Is your educational program approved by an external organisation?" #15 is one of the following answers ("Yes")

ID 743

16. Which organisations are responsible for external audits of naturopathic educational programs in your region? What types of external audits are carried out by each organisation?

	Organisation name	Type of Auditor					Audit undertaken			
		Government	Private	Professional Association	Accrediting body	Other	Governance/Quality assurance	Course content, delivery and assessment	Clinical processes	Fin[illegible]
1		○	○	○	○	○	☐	☐	☐	
2		○	○	○	○	○	☐	☐	☐	
3		○	○	○	○	○	☐	☐	☐	
4		○	○	○	○	○	☐	☐	☐	
5		○	○	○	○	○	☐	☐	☐	
6		○	○	○	○	○	☐	☐	☐	

LOGIC Hidden unless: Question "Is your educational program approved by an external organisation?" #15 is one of the following answers ("Yes")

ID 747

17. Where the answer above was OTHER audits, please explain scope of audit/s.

Overall influence of regulation on education

VALIDATION Min = 0 Max = 7

ID 915

18. In your opinion to what degree overall does **regulation** influence **naturopathic education** in your region?

None — Moderate — High

☐ Not Applicable

Comments

ID 934

19. Which organisations outside of the educational institution influence the delivery/content of naturopathic education in your region?

- [ ] National naturopathic professional association/s (provide name)
- [ ] Regional naturopathic professional association/s (provide name)
- [ ] Accreditation body (e.g. Council on Naturopathic Medicine Education)
- [ ] Regulatory board/s (provide name)
- [ ] Professional association/s for other health professions (e.g. medical doctors, nurses) (provide name/s)
- [ ] Third party funders (e.g. health insurance, Veteran's affairs) (provide details)
- [ ] National government department (provide details)
- [ ] Regional government department (provide details)
- [ ] Multinational body (e.g. European Commission)

ID 918

20. We would like to know more about the impact of specific organisations on characteristics of formal naturopathic education in your region. Please identify the degree, if at all, to which the following course characteristics are affected by various organisations. For example, what is the degree of influence of national professional associations upon the entry requirements for enrolment in a naturopathic education program?

	Admission requirements	Tuition fee schedule	Clinical supervisor/student ratio
Naturopathic educational institution	None<br>Low<br>Moderate<br>High<br>Unsure	None<br>Low<br>Moderate<br>High<br>Unsure	None<br>Low<br>Moderate<br>High<br>Unsure
National professional association(s)	None<br>Low<br>Moderate<br>High<br>Unsure	None<br>Low<br>Moderate<br>High<br>Unsure	None<br>Low<br>Moderate<br>High<br>Unsure
Regional professional association(s)	None<br>Low<br>Moderate<br>High<br>Unsure	None<br>Low<br>Moderate<br>High<br>Unsure	None<br>Low<br>Moderate<br>High<br>Unsure
Regulatory board(s)	None<br>Low<br>Moderate<br>High<br>Unsure	None<br>Low<br>Moderate<br>High<br>Unsure	None<br>Low<br>Moderate<br>High<br>Unsure

Third party funders	None<br>Low<br>Moderate<br>High<br>Unsure	None<br>Low<br>Moderate<br>High<br>Unsure	None<br>Low<br>Moderate<br>High<br>Unsure
National government department(s)	None<br>Low<br>Moderate<br>High<br>Unsure	None<br>Low<br>Moderate<br>High<br>Unsure	None<br>Low<br>Moderate<br>High<br>Unsure
Regional government department(s)	None<br>Low<br>Moderate<br>High<br>Unsure	None<br>Low<br>Moderate<br>High<br>Unsure	None<br>Low<br>Moderate<br>High<br>Unsure
Industry (e.g natural product/supplement manufacturers)	None<br>Low<br>Moderate<br>High<br>Unsure	None<br>Low<br>Moderate<br>High<br>Unsure	None<br>Low<br>Moderate<br>High<br>Unsure
Multi-national body (e.g. European Commission)	None<br>Low<br>Moderate<br>High<br>Unsure	None<br>Low<br>Moderate<br>High<br>Unsure	None<br>Low<br>Moderate<br>High<br>Unsure
Accreditation body (e.g. Council on Naturopathic Medicine Education)	None<br>Low<br>Moderate<br>High<br>Unsure	None<br>Low<br>Moderate<br>High<br>Unsure	None<br>Low<br>Moderate<br>High<br>Unsure

	Unsure	Unsure	Unsure

Comments

Organisations impacting on naturopathic education and course curriculum

Page description:
We would like to know more about the degree to which specific organisations impact upon naturopathic course curriculum.

For example, to what degree do national professional associations impact on the specific content included in naturopathic course curriculum?

ID 927

21. Please identify the degree, if at all, to which the following course curriculum characteristics are affected by the various organisations in your region.

Please use the scroll bar at the bottom of the table to access the full list of response options.

	Total course hours	Included content	Total program duration
Naturopathic educational institution	None<br>Low<br>Moderate<br>High<br>Unsure	None<br>Low<br>Moderate<br>High<br>Unsure	None<br>Low<br>Moderate<br>High<br>Unsure
	None<br>Low	None<br>Low	None<br>Low

National professional association(s)	Moderate<br>High<br>Unsure	Moderate<br>High<br>Unsure	Moderate<br>High<br>Unsure
Regional professional association(s)	None<br>Low<br>Moderate<br>High<br>Unsure	None<br>Low<br>Moderate<br>High<br>Unsure	None<br>Low<br>Moderate<br>High<br>Unsure
Regulatory board(s)	None<br>Low<br>Moderate<br>High<br>Unsure	None<br>Low<br>Moderate<br>High<br>Unsure	None<br>Low<br>Moderate<br>High<br>Unsure
Third party funder(s)	None<br>Low<br>Moderate<br>High<br>Unsure	None<br>Low<br>Moderate<br>High<br>Unsure	None<br>Low<br>Moderate<br>High<br>Unsure
National government department(s)	None<br>Low<br>Moderate<br>High<br>Unsure	None<br>Low<br>Moderate<br>High<br>Unsure	None<br>Low<br>Moderate<br>High<br>Unsure
Regional government department(s)	None<br>Low<br>Moderate<br>High<br>Unsure	None<br>Low<br>Moderate<br>High<br>Unsure	None<br>Low<br>Moderate<br>High<br>Unsure
	None	None	None

Industry (e.g natural product/supplement manufacturers)	Low<br>Moderate<br>High<br>Unsure	Low<br>Moderate<br>High<br>Unsure	Low<br>Moderate<br>High<br>Unsure
Multi-national body (e.g. European Commission)	None<br>Low<br>Moderate<br>High<br>Unsure	None<br>Low<br>Moderate<br>High<br>Unsure	None<br>Low<br>Moderate<br>High<br>Unsure
Accreditation body (e.g. Council on Naturopathic Medicine Education)	None<br>Low<br>Moderate<br>High<br>Unsure	None<br>Low<br>Moderate<br>High<br>Unsure	None<br>Low<br>Moderate<br>High<br>Unsure

ID 1136

22. Please identify the degree, if at all, to which the following course curriculum characteristics are affected by the various organisations in your region.

Please use the scroll bar at the bottom of the table to access the full list of response options.

	Delivery methods	Learning outcomes
Naturopathic educational institution	None<br>Low<br>Medium<br>High<br>Unsure	None<br>Low<br>Medium<br>High<br>Unsure
	None<br>Low	None<br>Low

National professional association(s)	Low<br>Medium<br>High<br>Unsure	Low<br>Medium<br>High<br>Unsure
Regional professional association(s)	None<br>Low<br>Medium<br>High<br>Unsure	None<br>Low<br>Medium<br>High<br>Unsure
Regulatory board(s)	None<br>Low<br>Medium<br>High<br>Unsure	None<br>Low<br>Medium<br>High<br>Unsure
Third party funder(s)	None<br>Low<br>Medium<br>High<br>Unsure	None<br>Low<br>Medium<br>High<br>Unsure
National government department(s)	None<br>Low<br>Medium<br>High<br>Unsure	None<br>Low<br>Medium<br>High<br>Unsure
Regional government department(s)	None<br>Low<br>Medium<br>High<br>Unsure	None<br>Low<br>Medium<br>High<br>Unsure
	None	None

Industry (e.g natural product/supplement manufacturers)	None<br>Low<br>Medium<br>High<br>Unsure	None<br>Low<br>Medium<br>High<br>Unsure
Multi-national body (e.g. European Commission)	None<br>Low<br>Medium<br>High<br>Unsure	None<br>Low<br>Medium<br>High<br>Unsure
Accreditation body (e.g. Council on Naturopathic Medicine Education)	None<br>Low<br>Medium<br>High<br>Unsure	None<br>Low<br>Medium<br>High<br>Unsure

Organisations affecting academic faculty

Page description:
We would like to know more about the impact of specific organisations upon the characteristics of academic faculty teaching in naturopathic programs. For example, to what degree do government departments impact upon the clinical experience required of clinical supervisors in your region?

ID 935

23. Please identify the degree, if at all, to which the following characteristics of ***classroom teachers*** are affected by the various organisations in your region.

	Minimum qualification of classroom teachers	Clinical experience of classroom teachers	Research experience or training of classroom teachers
	None	None	None

Naturopathic educational institution	Low<br>Moderate<br>High<br>Unsure	Low<br>Moderate<br>High<br>Unsure	Low<br>Medium<br>High<br>Unsure
National naturopathic association(s)	None<br>Low<br>Moderate<br>High<br>Unsure	None<br>Low<br>Moderate<br>High<br>Unsure	None<br>Low<br>Medium<br>High<br>Unsure
Regional naturopathic association(s)	None<br>Low<br>Moderate<br>High<br>Unsure	None<br>Low<br>Moderate<br>High<br>Unsure	None<br>Low<br>Medium<br>High<br>Unsure
Regulatory board(s)	None<br>Low<br>Moderate<br>High<br>Unsure	None<br>Low<br>Moderate<br>High<br>Unsure	None<br>Low<br>Medium<br>High<br>Unsure
Third party funder(s)	None<br>Low<br>Moderate<br>High<br>Unsure	None<br>Low<br>Moderate<br>High<br>Unsure	None<br>Low<br>Medium<br>High<br>Unsure
National government department(s)	None<br>Low<br>Moderate<br>High<br>Unsure	None<br>Low<br>Moderate<br>High<br>Unsure	None<br>Low<br>Medium<br>High<br>Unsure

Regional government department(s)	None<br>Low<br>Moderate<br>High<br>Unsure	None<br>Low<br>Moderate<br>High<br>Unsure	None<br>Low<br>Medium<br>High<br>Unsure
Industry (e.g natural product/supplement manufacturers)	None<br>Low<br>Moderate<br>High<br>Unsure	None<br>Low<br>Moderate<br>High<br>Unsure	None<br>Low<br>Medium<br>High<br>Unsure
Multi-national body (e.g. European Commission)	None<br>Low<br>Moderate<br>High<br>Unsure	None<br>Low<br>Moderate<br>High<br>Unsure	None<br>Low<br>Medium<br>High<br>Unsure
Accreditation body (e.g. Council on Naturopathic Medicine Education)	None<br>Low<br>Moderate<br>High<br>Unsure	None<br>Low<br>Moderate<br>High<br>Unsure	None<br>Low<br>Medium<br>High<br>Unsure

ID 1142

24. Please identify the degree, if at all, to which the following characteristics of ***clinical supervisors*** are affected by the various organisations in your region.

	Minimum qualifications of clinical supervisors	Clinical experience of clinical supervisors	Research experience or training of clinical supervisors
	None<br>Low	None<br>Low	None<br>Low

Naturopathic educational institution	Moderate<br>High<br>Unsure	Medium<br>High<br>Unsure	Medium<br>High<br>Unsure
National naturopathic association(s)	None<br>Low<br>Moderate<br>High<br>Unsure	None<br>Low<br>Medium<br>High<br>Unsure	None<br>Low<br>Medium<br>High<br>Unsure
Regional naturopathic association(s)	None<br>Low<br>Moderate<br>High<br>Unsure	None<br>Low<br>Medium<br>High<br>Unsure	None<br>Low<br>Medium<br>High<br>Unsure
Regulatory board(s)	None<br>Low<br>Moderate<br>High<br>Unsure	None<br>Low<br>Medium<br>High<br>Unsure	None<br>Low<br>Medium<br>High<br>Unsure
Third party funder(s)	None<br>Low<br>Moderate<br>High<br>Unsure	None<br>Low<br>Medium<br>High<br>Unsure	None<br>Low<br>Medium<br>High<br>Unsure
National government department(s)	None<br>Low<br>Moderate<br>High<br>Unsure	None<br>Low<br>Medium<br>High<br>Unsure	None<br>Low<br>Medium<br>High<br>Unsure
	None	None	None

Regional government department(s)	Low<br>Moderate<br>High<br>Unsure	Low<br>Medium<br>High<br>Unsure	Low<br>Medium<br>High<br>Unsure
Industry (e.g natural product/supplement manufacturers)	None<br>Low<br>Moderate<br>High<br>Unsure	None<br>Low<br>Medium<br>High<br>Unsure	None<br>Low<br>Medium<br>High<br>Unsure
Multi-national body (e.g. European Commission)	None<br>Low<br>Moderate<br>High<br>Unsure	None<br>Low<br>Medium<br>High<br>Unsure	None<br>Low<br>Medium<br>High<br>Unsure
Accreditation body (e.g. Council on Naturopathic Medicine Education)	None<br>Low<br>Moderate<br>High<br>Unsure	None<br>Low<br>Medium<br>High<br>Unsure	None<br>Low<br>Medium<br>High<br>Unsure

The structure of health practitioner regulation

LOGIC Show/hide trigger exists.

ID 1148

25. Does regulation of *any* health profession (e.g. medical doctors/nurses/naturopaths etc.) exist in your region?

- Yes
- No
- Unsure

VALIDATION Must be numeric

LOGIC Hidden unless: Question "Does regulation of *any* health profession (e.g. medical doctors/nurses/naturopaths etc.) exist in your region?" #25 is one of the following answers ("Yes")

ID 1023

26. How many health professions are regulated in your region?

LOGIC Hidden unless: Question "Does regulation of *any* health profession (e.g. medical doctors/nurses/naturopaths etc.) exist in your region?" #25 is one of the following answers ("Yes")

ID 1054

27. Please indicate if any of the following health PROFESSIONS are regulated in your region and if so what type of regulation is in place:

	No regulation	Protection of title	Defined scope of practice	Other
Acupuncturists	☐	☐	☐	☐
Ayurvedic practitioners	☐	☐	☐	☐
Chiropractors	☐	☐	☐	☐
Complementary medicine providers (as a group)	☐	☐	☐	☐
Dieticians	☐	☐	☐	☐
Herbalists	☐	☐	☐	☐
Homeopaths	☐	☐	☐	☐
Integrative medicine practitioners (MDs using complementary medicine in their practice)	☐	☐	☐	☐
Medical doctors	☐	☐	☐	☐
Naturopaths/Naturopathic doctors	☐	☐	☐	☐
Nurses	☐	☐	☐	☐
Nutritionists	☐	☐	☐	☐
Osteopaths	☐	☐	☐	☐
Pharmacists	☐	☐	☐	☐
Traditional Chinese medicine practitioners	☐	☐	☐	☐
Traditional healers	☐	☐	☐	☐

The structure of naturopathic regulation

Page description:
The following questions relate specifically to the practice of naturopathic profession in your region. Information pertaining to natural health products will be collected later in this survey.

LOGIC Hidden unless: Question "Naturopaths/Naturopathic doctors" is one of the following answers ("Protection of title","Defined scope of practice","Other")

ID 1206

28. In the previous question, you have identified that some form of regulation exists for the practice of naturopathy in your region.

When was the regulation of naturopathy first implemented in your region?

1949 or earlier
1950
1951
1952
1953
1954
1955
1956
1957
1958
1959
1960
1961
1962
1963
1964
1965
1966
1967
1968
1969
1970

1971
1972
1973
1974
1975
1976
1977
1978
1979
1980
1981
1982
1983
1984
1985
1986
1987
1988
1989
1990
1991
1992
1993
1994
1995
1996
1997
1998
1999
2000
2001
2002
2003
2004
2005
2006
[illegible]

2007
2008
2009
2010
2011
2012
2013
2014
2015
2016
2017
2018

LOGIC Hidden unless: Question "Naturopaths/Naturopathic doctors" is one of the following answers ("Protection of title","Defined scope of practice","Other")

ID 1124

29. Are there any other legislative requirements outside of the health professional regulation identified on the previous page which directly impact on the practice of naturopathy in your region?

- Yes
- No
- Unsure

LOGIC Hidden unless: Question "Naturopaths/Naturopathic doctors" is one of the following answers ("Protection of title","Defined scope of practice","Other")

ID 1114

30. What is the jurisdiction covered by naturopathic registration in your region

- ○ National
- ○ Regional
- ○ Other - please specify

*

LOGIC Hidden unless: Question "Naturopaths/Naturopathic doctors" is one of the following answers ("Protection of title","Defined scope of practice","Other")

ID 987

31. Which of the following determines the practices included within the regulation for naturopathic practitioners in your region?

- ☐ Inclusion in naturopathic curriculum
- ☐ Regulatory guidelines and restrictions
- ☐ Current naturopathic practice

ID 942

32. Is your organisation in support of regulation of the naturopathic profession in your region?

- Yes
- No
- The organisation's position on this issue is undecided
- I am unsure of my organisation's position on this issue

LOGIC Show/hide trigger exists.

ID 943

33. Does your organisation have a formal policy or position statement with regards to regulation of the naturopathic profession in your region?

- Yes
- No
- Unsure

LOGIC Hidden unless: Question "Does your organisation have a formal policy or position statement with regards to regulation of the naturopathic profession in your region?" #33 is one of the following answers ("Yes")

ID 944

34. What is the reason for the formal position on regulation of the naturopathic profession held by your organisation?

LOGIC Hidden unless: Question "Does your organisation have a formal policy or position statement with regards to regulation of the naturopathic profession in your region?" #33 is one of the following answers ("No")

ID 945

35. What is the reason your organisation does not hold a formal position on regulation of the naturopathic profession?

Restricted or controlled acts

ID 1181

36. Please identify which of the following are restricted or controlled acts for naturopaths in your region (select all that apply):

	Full access available to naturopaths	Limited access available to naturopaths	Permitted but not included in all curriculum	Not allowed	Unsure
Ordering laboratory/pathology tests and scans	○	○	○	○	○
Obstetrics/maternity care	○	○	○	○	○
Diagnosis of health conditions	○	○	○	○	○
Taking blood samples	○	○	○	○	○
Intravenous administration	○	○	○	○	○
Minor surgery	○	○	○	○	○
Acupuncture	○	○	○	○	○
Cervical manipulation	○	○	○	○	○
General manipulation	○	○	○	○	○
Prescription of pharmaceuticals	○	○	○	○	○
Prescription of naturopathic treatments	○	○	○	○	○
Sale of naturopathic products within a naturopathic clinic	○	○	○	○	○
General physical exam	○	○	○	○	○
Ordering of non-conventional laboratory tests (e.g. urine/hair/stool analysis)	○	○	○	○	○

ID 1150

37. Please identify which of the following activities are available to naturopaths in your region (select all that apply):

	Full access available to naturopaths	Limited access available to naturopaths	Not allowed	Not applicable	Unsure
Private health insurance reimbursement	☐	☐	☐	☐	☐
Public health (government funded) reimbursement	☐	☐	☐	☐	☐
Medical integration (hospital privileges)	☐	☐	☐	☐	☐

Factors impacting naturopathic regulation

ID 946

38. Please identify the degree, if at all, to which the following factors impact on regulation in your region.

	None	Low	Moderate	High	Unsure
Regulation or scope of other health practitioners	○	○	○	○	○
Research evidence supporting naturopathy	○	○	○	○	○
Attitude of decision makers	○	○	○	○	○
Naturopathic regulation in other regions	○	○	○	○	○
Desire for regulation amongst naturopaths in your region	○	○	○	○	○
Number of hours in a naturopathic qualification	○	○	○	○	○
Naturopathic curriculum content	○	○	○	○	○
Level of naturopathic qualification awarded (e.g. Degree vs Diploma)	○	○	○	○	○
Opinion of other health practitioner groups (including medical doctors)	○	○	○	○	○
Number of naturopathic practitioners in your region	○	○	○	○	○
Professional formation of representative organisations	○	○	○	○	○
Financial resources for lobbying	○	○	○	○	○
Skills and experience in lobbying	○	○	○	○	○
Professional unity	○	○	○	○	○
Existing legislation	○	○	○	○	○

Impact of organisations on naturopathic standards

Page description:
We would like to know more about the impact of specific organisations on the management of safe, quality naturopathic practice in your region.

ID 1034

39. Please identify the degree, if at all, to which the following are affected by different organisations

	Setting standards/practice guidelines	Enforcing standards/practice guidelines	Educating practitioners about standards/practice guidelines
Naturopathic educational institution	None<br>Low<br>Moderate<br>High<br>Unsure<br>Not applicable	None<br>Low<br>Moderate<br>High<br>Unsure<br>Not applicable	None<br>Low<br>Moderate<br>High<br>Unsure<br>Not applicable
National professional association(s)	None<br>Low<br>Moderate<br>High<br>Unsure<br>Not applicable	None<br>Low<br>Moderate<br>High<br>Unsure<br>Not applicable	None<br>Low<br>Moderate<br>High<br>Unsure<br>Not applicable
Regional professional association(s)	None<br>Low<br>Moderate<br>High<br>Unsure<br>Not applicable	None<br>Low<br>Moderate<br>High<br>Unsure<br>Not applicable	None<br>Low<br>Moderate<br>High<br>Unsure<br>Not applicable
Regulatory board(s)	None<br>Low<br>Moderate<br>High<br>Unsure<br>Not applicable	None<br>Low<br>Moderate<br>High<br>Unsure<br>Not applicable	None<br>Low<br>Moderate<br>High<br>Unsure<br>Not applicable
Third party funder(s)	None<br>Low<br>Moderate	None<br>Low<br>Moderate	None<br>Low<br>Moderate

Third party funder(s)	High<br>Unsure<br>Not applicable	High<br>Unsure<br>Not applicable	High<br>Unsure<br>Not applicable
National government department(s)	None<br>Low<br>Moderate<br>High<br>Unsure<br>Not applicable	None<br>Low<br>Moderate<br>High<br>Unsure<br>Not applicable	None<br>Low<br>Moderate<br>High<br>Unsure<br>Not applicable
Regional government department(s)	None<br>Low<br>Moderate<br>High<br>Unsure<br>Not applicable	None<br>Low<br>Moderate<br>High<br>Unsure<br>Not applicable	None<br>Low<br>Moderate<br>High<br>Unsure<br>Not applicable
Industry (e.g natural product/supplement manufacturers)	None<br>Low<br>Moderate<br>High<br>Unsure<br>Not applicable	None<br>Low<br>Moderate<br>High<br>Unsure<br>Not applicable	None<br>Low<br>Moderate<br>High<br>Unsure<br>Not applicable
Multi-national body (e.g. European Commission)	None<br>Low<br>Moderate<br>High<br>Unsure<br>Not applicable	None<br>Low<br>Moderate<br>High<br>Unsure<br>Not applicable	None<br>Low<br>Moderate<br>High<br>Unsure<br>Not applicable
Accreditation body (e.g. Council on [illegible]	None<br>Low<br>Moderate<br>[illegible]	None<br>Low<br>Moderate<br>[illegible]	None<br>Low<br>Moderate<br>[illegible]

Naturopathic Medicine Education)	High<br>Unsure<br>Not applicable	High<br>Unsure<br>Not applicable	High<br>Unsure<br>Not applicable

Comments

ID 1173

40. Please identify the degree, if at all, to which the following are affected by the various organisations in your region.

	Requesting changes to standards/practice guidelines	Lobbying for naturopathic regulation in your region
Naturopathic educational institution	None<br>Low<br>Moderate<br>High<br>Unsure<br>Not applicable	None<br>Low<br>Moderate<br>High<br>Unsure<br>Not applicable
National professional association(s)	None<br>Low<br>Moderate<br>High<br>Unsure<br>Not applicable	None<br>Low<br>Moderate<br>High<br>Unsure<br>Not applicable
	None<br>Low<br>Moderate	None<br>Low<br>Moderate

Regional professional association(s)	[illegible]<br>High<br>Unsure<br>Not applicable	[illegible]<br>High<br>Unsure<br>Not applicable
Regulatory board(s)	None<br>Low<br>Moderate<br>High<br>Unsure<br>Not applicable	None<br>Low<br>Moderate<br>High<br>Unsure<br>Not applicable
Third party funder(s)	None<br>Low<br>Moderate<br>High<br>Unsure<br>Not applicable	None<br>Low<br>Moderate<br>High<br>Unsure<br>Not applicable
National government department(s)	None<br>Low<br>Moderate<br>High<br>Unsure<br>Not applicable	None<br>Low<br>Moderate<br>High<br>Unsure<br>Not applicable
Regional government department(s)	None<br>Low<br>Moderate<br>High<br>Unsure<br>Not applicable	None<br>Low<br>Moderate<br>High<br>Unsure<br>Not applicable
Industry (e.g natural product/supplement [illegible]	None<br>Low<br>[illegible]	None<br>Low<br>[illegible]

Industry (e.g natural product/supplement manufacturers)	Moderate<br>High<br>Unsure<br>Not applicable	Moderate<br>High<br>Unsure<br>Not applicable
Multi-national bodies (e.g. European Commission)	None<br>Low<br>Moderate<br>High<br>Unsure<br>Not applicable	None<br>Low<br>Moderate<br>High<br>Unsure<br>Not applicable
Accreditation body (e.g. Council on Naturopathic Medicine Education)	None<br>Low<br>Moderate<br>High<br>Unsure<br>Not applicable	None<br>Low<br>Moderate<br>High<br>Unsure<br>Not applicable

Comments

ID 1042

41. Are there any other organisations that overtly SUPPORT naturopathic regulation in your region? Please list each organisation and the details of their position on the issue of naturopathic regulation.

ID 1043

42. Are there any other organisations that overtly OPPOSE naturopathic regulation in your region? Please list each organisation and the details of their position on the issue of naturopathic regulation.

Naturopathic registration requirements

Page entry logic:
This page will show when: Question "Naturopaths/Naturopathic doctors" is one of the following answers ("Protection of title","Defined scope of practice","Other")

LOGIC Show/hide trigger exists.

ID 969

43. What is required for a naturopath to be registered in your region?

- ☐ Direct entry based on completion of specific qualifications, but no requirement to graduate from a naturopathic educational institution
- ☐ Direct entry based on achieving qualification/graduation from a naturopathic institution, but no need to write a board exam
- ☐ Entry based on achieving qualification/graduation from a naturopathic institution, plus the need to write a board exam
- ☐ Entry based on the successful completion of a board exam, without any other specific qualifications
- ☐ Other - please specify

*

LOGIC Hidden unless: Question "What is required for a naturopath to be registered in your region?" #43 is one of the following answers ("Entry based on achieving qualification/graduation from a naturopathic institution, plus the need to write a board exam")

ID 970

44. Who administers the board exam used for naturopathic regulation in your region?

- Government department or agency
- Delegated authority on behalf of the government
- Independent academic accreditation agency
- Naturopathic educational institution
- Naturopathic organisation independent of the naturopathic educational institution
- Other - Please specify

*

LOGIC Hidden unless: Question "What is required for a naturopath to be registered in your region?" #43 is one of the following answers ("Entry based on achieving qualification/graduation from a naturopathic institution, plus the need to write a board exam")

ID 971

45. How does an applicant qualify to sit a board exam?

- No entry requirements
- Entry based on experience or equivalence qualifications
- Direct entry based on qualification (no institutional accreditation required)
- Direct entry based on qualification from an accredited institution only

Naturopathic regulatory bodies

Page entry logic:
This page will show when: Question "Naturopaths/Naturopathic doctors" is one of the following answers ("Protection of title","Defined scope of practice","Other")

ID 1044

46. Who is responsible for approving regulation of naturopathy in your region?

- A government official (e.g. the Minister of Health)
- A committee or board
- An existing regulatory body for other health professions
- An existing regulatory body for naturopathy
- Other - Please specify

*

ID 1045

47. Please provide more information about the regulatory body for naturopathy in your region?

Is there a regulatory body specific for naturopathy in your region? (i.e. does the body only focus on naturopathy or does it encompass a number of health professions?)

- Yes
- No
- Unsure

Which of the following best describes the regulatory body for naturopathy in your region?

- A standalone organisation with no government affiliation
- An independent organisation affiliated with the government

An independent organisation affiliated with the government

- An independent organisation affiliated with a professional association
- A government organisation
- A professional association

Is naturopathic regulation their sole purpose? (i.e. do they only oversee regulation of naturopathy or do they undertake other activities for the naturopathic profession)

- Yes
- No
- Unsure

If no, please explain

What is the composition of the body, committee or board primarily responsible for decisions related to naturopathic regulation in your region? Select all that apply.

- naturopaths/naturopathic practitioners
- public/community representatives
- public servants
- government officials
- independent representatives of other health professions
- representatives of other health professional bodies (e.g. professional associations)

☐ representatives of naturopathic professional associations

☐ representatives of naturopathic educational institutions

Regulatory efforts

Page entry logic:
This page will show when: Question "Naturopaths/Naturopathic doctors" is one of the following answers ("No regulation")

ID 1055

48. Which of the following reflects the activities and achievements in your region with regards to achieving regulation of the naturopathic profession? Please provide details where possible.

	Attempted			Please provide details
	Yes	No	Not applicable	
Developing a plan to be implemented at a later date which focuses on achieving regulation of the naturopathic profession	○	○	○	
Actively work to achieve regulation of the naturopathic profession is underway	○	○	○	

Timelines for attaining regulation of the naturopathic profession have been set	○	○	○	
Obstacles to attaining regulation of the naturopathic profession have been identified	○	○	○	
Establishment of a professional association for the naturopathic profession	○	○	○	
Initial steps for regulation of the naturopathic profession are underway	○	○	○	
Developing a plan to be implemented at a later date which focuses on enhancing or improving regulation of the naturopathic profession	○	○	○	

VALIDATION Min = 0 Max = 7

ID 1058

49. In your opinion, to what degree would assistance in the following areas progress attempts to achieve naturopathic regulation in your region? Please provide any additional details in the comments box below

	Not at all	Somewhat	Substantially
Dedicated human resources			
Training for local naturopathic leaders			
Advice on current regulatory efforts			
Positive media for the profession			
Support in attaining professional unity in regulatory efforts			

Comments

VALIDATION Min = 0 Max = 7

ID 1059

50. To what degree is involvement in the World Naturopathic Federation seen as support for regulation in your region?

None | Moderate | High

Comments

Overall impact of naturopathic education on regulation

VALIDATION Min = 0 Max = 7

ID 916

51. In your opinion to what degree does naturopathic *education* influence *regulation* of naturopathy in your region?

None | Moderate | High

☐ Not Applicable

Comments

LOGIC Show/hide trigger exists.

ID 1061

52. Is there regulation of natural health products in your region?

- ○ Yes
- ○ No
- ○ In progress
- ○ Unsure

LOGIC Hidden unless: Question "Is there regulation of natural health products in your region?" #52 is one of the following answers ("Yes")

ID 1062

53. Please provide additional information about the regulation of natural health products in your region:

What is the name of the Ministry, Directorate or Organisation responsible for managing regulation of natural health products in your region?

What is the name of the relevant regulation/legislation?

Which natural health products are affected by the regulation?

- ○ botanical medicines

- nutraceuticals
- homeopathics
- Other - please specify

What does the regulation/legislation cover?

- ☐ ingredient restrictions
- ☐ quality of substances
- ☐ efficacy
- ☐ advertising

What is the access to the following products in line with the regulation/legislation?

	Botanical medicine	Nutraceuticals	Homeopathics	Other
Restricted and requires a prescription	☐	☐	☐	☐
Access to health professionals only	☐	☐	☐	☐
Restricted but available to consumers (i.e. behind the counter)	☐	☐	☐	☐
Freely available to consumers (e.g. over the counter)	☐	☐	☐	☐
Not available at all	☐	☐	☐	☐

Are there any natural health products that have specific restrictions or limitations or that appear on government schedules – i.e., an herb that may be restricted or a nutraceutical above a specific dose. Please list as many relevant examples as possible or please indicate if you are unsure of specific examples.

Reimbursement of costs associated with naturopathic consultations and treatments

ID 1024

54. What options are available to patients in your region to cover the costs of naturopathic consultations and treatments?

	Consultation costs	Prescriptions for naturopathic products (e.g. supplements, herbal medicine, homeopathy)	In-office treatments (e.g. acupuncture, manual therapy)
Direct payment by patient and no reimbursement from third party payer (insurance company)	☐	☐	☐
Government funded/public health reimbursement	☐	☐	☐
Private health insurance reimbursement	☐	☐	☐
Other third party funder reimbursement (Veteran's affairs, employer-funded workplace injury insurance)	☐	☐	☐
Not applicable	☐	☐	☐
Enter another option	☐	☐	☐
Enter another option	☐	☐	☐

ID 1029

55. Which of the following factors impact upon patient access to reimbursement for costs associated with naturopathic services in your region? Select all that apply

- [ ] Practitioner must have malpractice insurance
- [ ] Practitioner must have registered title/number from their regulatory body
- [ ] Patient characteristics (veterans, workplace injury, elderly)
- [ ] Diagnosed disease/condition
- [ ] Level of practitioner education/qualification
- [ ] Referral from a medical practitioner
- [ ] Type of therapies/treatments provided by the practitioner
- [ ] Other - Please specify
- [ ] Other - Please specify
- [ ] Not applicable

Reimbursement of costs associated with naturopathic care

VALIDATION Min = 0 Max = 7

ID 1022

56. In your opinion to what degree do you think reimbursement of costs associated with naturopathic care is tied to regulation in your region/country?

None | Moderate | High

☐ Not Applicable

Comments

VALIDATION Min = 0 Max = 7

ID 1032

57. In your opinion, to what degree do you think reimbursement of costs associated with naturopathic care influences the integration of naturopathy into the wider healthcare system in your region/country?

None | Moderate | High

☐ Not Applicable

Comments

VALIDATION Min = 0 Max = 7

ID 1033

58. In your opinion to what degree do you think reimbursement of costs associated with naturopathic care is tied to the level of use of naturopathy in your region/country?

None — Moderate — High

☐ Not Applicable

Comments

Participation in future stages of this research project

ID 1172

59. This survey represents the first stage of a larger research project aimed at understanding the factors which impact upon the practice of naturopathy around the world.

The research team would like to contact you in the future to clarify any information you have provided in this survey to inform the next stage of the project. Do you agree to being contacted for this purpose?

- ○ Yes
- ○ No

Thank You!

LOGIC Hidden unless: (Question "Which of the following best describes your organisation?" #1 is one of the following answers ("Educational institution with a specific program that trains naturopathic practitioners","Naturopathic professional association","Organisation that regulates the practice of naturopathy") AND Question "Which of the following criteria describes the naturopathic training/education programme delivered or supported by your institution? Please select ALL that apply." #2 is one of the following answers ("At least 1500 contact/teaching hours (or equivalent)","Minimum of 2 years in length","Minimum of 400 hours of supervised clinical practicum in a residential clinic setting"))

ID 1

Thank you for completing this questionnaire.

The information that you have provided will assist the global professional mapping of naturopathy. A report of this survey will be shared with your organisation in the near future and is likely to assist advancement of the naturopathic profession internationally.

LOGIC Hidden unless: ((Question "Which of the following best describes your organisation?" #1 is one of the following answers ("None of the above") OR Question "Which of the following criteria describes the naturopathic training/education programme delivered or supported by your institution? Please select ALL that apply." #2 is one of the following answers ("None of the above")) OR Question "Which of the following criteria describes the naturopathic training/education programme delivered or supported by your institution? Please select ALL that apply." #2 is not exactly equal to ("At least 1500 contact/teaching hours (or equivalent)","Minimum of 2 years in length","Minimum of 400 hours of supervised clinical practicum in a residential clinic setting"))

ID 11

Thank you for contributing to this survey.

Unfortunately, your organisation does not meet the criteria for inclusion in this survey. If you have any further queries please contact the research team at Jillian.M.Dunn@uts.edu.au

Confirmation Email

To: Jill Dunn (jilldunn@iconz.co.nz)

From: SurveyGizmo (notifications@surveygizmo.com)

Subject: New Response Notification

Confirmation Email

To: [question("value"), id="24"] [question("value"), id="25"] ([question("value"), id="34"])

From: SurveyGizmo (notifications@surveygizmo.com)

Subject: Your survey response: Mapping the regulatory and educational landscape of naturopathy
