# Supplementary material for: Characteristics of global naturopathic education, regulation, and practice frameworks: results from an international survey
Source: BMC Complement Med Ther. 2021 Feb 18;21:67. doi: 10.1186/s12906-021-03217-1 (PMC7893718; doi:10.1186/s12906-021-03217-1)
Supplement: Supplementary file 2 — Additional file 2. [file 12906_2021_3217_MOESM2_ESM.docx]

# **Supplementary information -** Characteristics of global naturopathic education, regulation, and practice frameworks: results from an international survey

Data collection phase by country Nov 2016 – June 2019

| **Country** | **Collection time** |
| --- | --- |
| Australia | Jan 2017 – Oct 2018 |
| Belgium | March 2017 |
| Brazil | Feb 2017 – May 2019 |
| Canada | Dec 2016 – April 2017 |
| Chile | June 2018 |
| Congo DR | June 2018 |
| Cyprus | Aug 2018 |
| Czech Republic | Aug 2018 – Sept 2018 |
| Egypt | June 2019 |
| France | Dec 2016 – May 2017 |
| Germany | Nov 2018 |
| Hong Kong | Aug 2018 |
| Italy | July 2018 |
| Mexico | Feb 2017 |
| Nepal | Dec 2016 |
| New Zealand | Dec 2016 – Feb 2017 |
| Peru | July 2018 |
| Portugal | Nov 2016 |
| Slovenia | Feb 2017 |
| South Africa | Aug 2018 |
| Spain | Feb 2017 |
| Sweden | Dec 2016 |
| Switzerland | May 2018 |
| United Kingdom | Dec 2016 – March 2017 |
| USA | Dec 2016 – Aug 2018 |
| Puerto Rico | April 2017 – Nov 2018 |
| Uruguay | April 2017 – May 2017 |
| Venezuela | Dec 2016 |
| Zambia | Jan 2017 |
